# Sex-stratified associations of TyG index and HOMA-IR changes with incident type 2 diabetes

**DOI:** 10.1038/s41598-026-52072-y

**Published:** 2026-05-08

**Authors:** Sojin Kim, Sujeong Shin, Yoosoo Chang, Eunju Sung, Jae-Heon Kang

**Affiliations:** 1 Human-Centered Artificial Intelligence Research Institute, Ewha Womans University, Seoul, South Korea; 2 Department of Family Medicine, Kangbuk Samsung Hospital, Sungkyunkwan University, School of Medicine, 29, Saemunan-ro, Jongno-gu, Seoul, 03181 Republic of Korea; 3 Center for Cohort Studies, Total Healthcare Center, Kangbuk Samsung Hospital, Sungkyunkwan University School of Medicine, Seoul, South Korea; 4 Department of Occupational and Environmental Medicine, Kangbuk Samsung Hospital, Sungkyunkwan University School of Medicine, Seoul, South Korea; 5 Department of Clinical Research Design and Evaluation, SAIHST, Sungkyunkwan University, Seoul, South Korea

**Keywords:** Triglycerides, Glucose, Homeostasis model assessment, Type 2 diabetes mellitus, Insulin resistance, Diseases, Endocrinology, Health care, Medical research, Risk factors

## Abstract

Early identification of individuals at risk of type 2 diabetes is important in young and middle-aged adults. Although the homeostatic model assessment of insulin resistance (HOMA-IR) is widely used, its limited clinical practicality has increased interest in the triglyceride–glucose (TyG) index as a simpler surrogate marker of insulin resistance. However, the predictive values of longitudinal changes in these indices remain unclear. We analyzed data from 271,592 Korean adults aged 18–49 years who underwent at least two health screening examinations. Changes in the TyG index and HOMA-IR were categorized into sex-specific quintiles. Incident type 2 diabetes was defined as fasting glucose ≥ 126 mg/dL, HbA1c ≥ 6.5%, or initiation of antidiabetic medication. Hazard ratios were estimated using the Cox proportional hazard models. During the follow-up period, 3,841 men and 1,069 women developed type 2 diabetes. Greater increases in the TyG index were consistently associated with a higher risk of diabetes across quintiles in both sexes. The associations with HOMA-IR changes were more variable across analytical models, particularly in the lower quintiles. These findings suggest that monitoring changes in both indices may provide additional information for diabetes risk assessment, with the TyG index showing more consistent associations across models in our study. Further studies in diverse populations are needed to confirm these observations.

**Supplementary Information:**

The online version contains supplementary material available at 10.1038/s41598-026-52072-y.

## Introduction

The global incidence of type 2 diabetes has progressively increased over the recent decades, accompanied by a concurrent rise in prediabetes^1,2^. This trend is especially worrisome for young and middle-aged adults, as early metabolic issues in this group could lead to extended exposure to hyperglycemia and its associated complications over a lifetime^3–5^. Therefore, identifying high-risk individuals within this population is a public health priority, as effective prevention strategies can yield greater long-term benefits when implemented early in life^6,7^. Sex-specific differences in insulin resistance and metabolic risk have been well documented, yet whether the predictive implications of longitudinal changes in TyG index and HOMA-IR differ by sex remains unclear^8,9^.

Insulin resistance (IR), a key mechanism linking individual susceptibility and lifestyle factors in the development of type 2 diabetes, is commonly assessed using the homeostasis model assessment of insulin resistance (HOMA-IR)^10,11^. However, its clinical utility is limited by the requirement for fasting insulin measurements, which lack standardization and are not routinely performed^12,13^. The triglyceride-glucose (TyG) index, derived from fasting triglyceride and glucose levels, has emerged as a simple surrogate for IR, showing predictive performance comparable to or greater than HOMA-IR^14–16^. Despite their utility, both the HOMA-IR and TyG indices are derived from laboratory values that fluctuate with metabolic and lifestyle changes. Although several studies have examined changes in the TyG index or HOMA-IR in relation to cardiovascular outcomes, mortality, or diabetes risk, most have involved middle-aged or older adults, and some have not excluded individuals with pre-existing diabetes mellitus. Moreover, large-scale prospective studies evaluating changes in both indices among metabolically healthy young and middle-aged adults are still limited^17–19^. Furthermore, while prior studies have predominantly assessed TyG index and HOMA-IR as static measurements, the incremental predictive value of longitudinal changes in these indices for the prediction of incident type 2 diabetes remains to be established^20,21^. This question is particularly relevant to young and middle-aged adults, in whom lifestyle modifications and improved fitness can influence the trajectories of insulin resistance^5^, and whether the predictive implications of these changes differ by sex remains unclear.

In this context, we conducted a large-scale cohort study of young and middle-aged Korean adults to investigate sex-stratified associations between changes in the TyG index and HOMA-IR with incident type 2 diabetes. We hypothesized that improvements in insulin resistance–related indices would be associated with a lower risk of type 2 diabetes, with the TyG index demonstrating more consistent predictive performance than HOMA-IR.

## Result

### Baseline characteristics

Table 1 summarizes the baseline characteristics of the study population according to sex and incident type 2 diabetes mellitus status. Among 141,059 men and 130,553 women, incident type 2 diabetes occurred in 3,841 men (2.72%) and 1,069 women (0.82%), with a higher incidence in men than that in women. Men with type 2 diabetes were slightly older and had higher BMI, waist circumference, blood pressure, HbA1c, insulin, fasting blood glucose, triglyceride, and HOMA-IR levels than men without type 2 diabetes. Similar patterns were observed in women with type 2 diabetes, who exhibited higher metabolic risk factors than their non-type 2 diabetes counterparts. In both sexes, individuals with type 2 diabetes had a higher prevalence of diabetes in their family history.

**Table 1:** Baseline characteristics of the study population by sex and type 2 diabetes status.

Variables	Men			Women		
	Total	Type 2 diabetes	Non-Type 2 diabetes	Total	Type 2 diabetes	Non-type 2 diabetes
N	141,059	3,841	137,218	130,533	1,069	129,464
Age, years	34.11 ± 5.57	35.08 ± 5	34.08 ± 5.59	33.42 ± 5.7	33.99 ± 5.39	33.41 ± 5.7
Alcohol intake (%)	34,263 (24.94)	1,227 (31.94)	33,036 (24.08)	16,666 (14.04)	165 (15.43)	16,501 (12.75)
Regular physical activity (%)	21,519 (15.35)	455 (11.85)	21,064 (15.35)	11,791 (9.12)	122 (11.41)	11,669 (9.01)
Smoking status (%)						
Never	52,289 (37.52)	807 (21.01)	51,482 (37.52)	115,134 (91.24)	889 (83.16)	114,245 (88.24)
Former	43,687 (31.35)	1,371 (35.69)	42,316 (30.84)	7,751 (6.14)	89 (8.33)	7,662 (5.92)
Current	43,375 (31.13)	1,594 (41.5)	41,781 (30.45)	3,302 (2.62)	29 (2.71)	3,273 (2.53)
Education: University or more (%)	107,678 (78.14)	2,667 (69.44)	105,011 (76.53)	87,013 (68.59)	485 (45.37)	86,528 (66.84)
BMI, kg/m^2^	24.63 ± 3.05	26.66 ± 3.41	24.57 ± 3.02	21.35 ± 3.01	24.9 ± 4.35	21.32 ± 2.98
Waist circumference, cm	85.72 ± 8.2	91.06 ± 8.62	85.57 ± 8.14	73.99 ± 7.99	83.49 ± 10.43	73.92 ± 7.92
SBP, mmHg	113.93 ± 10.87	118.77 ± 11.76	113.8 ± 10.81	101.35 ± 9.96	107.22 ± 12.35	101.3 ± 9.92
DBP, mmHg	72.02 ± 8.87	76.39 ± 9.6	71.9 ± 8.82	64.73 ± 7.73	68.01 ± 9.39	64.7 ± 7.71
Total cholesterol, mg/dL	195.92 ± 33.88	207.24 ± 37.51	195.6 ± 33.72	183.46 ± 30.39	194.09 ± 32.47	183.37 ± 30.36
HbA1c, %	5.45 ± 0.25	5.74 ± 0.29	5.44 ± 0.25	5.4 ± 0.27	5.75 ± 0.3	5.39 ± 0.26
Insulin, uIU/mL	6.93 ± 4.09	8.93 ± 4.89	6.87 ± 4.05	6.07 ± 3.56	9.06 ± 5.39	6.05 ± 3.53
eGFR, mL/min/1.73m^2^	102.19 ± 12.83	101.71 ± 13.06	102.2 ± 12.82	112 ± 11.75	111.77 ± 11.52	112 ± 11.75
Uric acid, mg/dL	6.32 ± 1.23	6.68 ± 1.33	6.31 ± 1.23	4.3 ± 0.9	4.78 ± 1.09	4.3 ± 0.9
FBG, mg/dL	93.85 ± 7.96	100.9 ± 10.18	93.66 ± 7.8	89.7 ± 7.5	98.45 ± 10.51	89.63 ± 7.42
Triglycerides, mg/dL	125.25 ± 83.43	175.95 ± 125.06	123.83 ± 81.51	77.82 ± 40.62	114.36 ± 69.82	77.52 ± 40.16
TyG index						
1st	8.52 ± 0.56	8.93 ± 0.57	8.51 ± 0.56	8.06 ± 0.45	8.49 ± 0.55	8.05 ± 0.44
2nd	8.6 ± 0.56	9.02 ± 0.58	8.59 ± 0.56	8.11 ± 0.46	8.59 ± 0.55	8.11 ± 0.45
HOMA-IR						
1st	1.63 ± 1.03	2.26 ± 1.31	1.62 ± 1.02	1.37 ± 0.87	2.24 ± 1.4	1.36 ± 0.86
2nd	1.81 ± 1.14	2.62 ± 1.5	1.78 ± 1.12	1.47 ± 0.95	2.57 ± 1.61	1.46 ± 0.94
History of hypertension (%)	10,234 (7.26)	569 (14.81)	9,665 (7.04)	1,563 (1.2)	41 (3.84)	1,522 (1.18)
Usage of lipid-lowering medication (%)	2,098 (1.49)	111 (2.89)	1,987 (1.45)	432 (0.33)	15 (1.4)	417 (0.32)
Family history of diabetes (%)	15,534 (11.01)	777 (20.23)	14,757 (10.75)	17,828 (13.66)	304 (28.44)	17,524 (13.54)
Menopause (%)				1,928 (1.56)	1,904 (1.47)	24 (2.25)

BMI, body mass index; DBP, diastolic blood pressure; eGFR, estimated glomerular filtration rate; FBG, fasting blood glucose; HbA1c, glycated hemoglobin; HOMA-IR, homeostasis model assessment of insulin resistance; SBP, systolic blood pressure; TyG index, triglyceride-glucose index; Q, quintile.

Data are expressed as mean $\pm$ standard deviation or numbers (percentages).

One-way analysis of variance (ANOVA) was used for continuous variables, and the chi-square test was used for categorical variables.

Alcohol intake was defined as ≥ 20 g/day for men and ≥ 10 g/day for women. Regular physical activity was defined as vigorous exercise $\ge$ 3 times per week.

Supplementary Table S1 presents the quintile distribution of changes in the TyG index and HOMA-IR according to the sex and type 2 diabetes status of the participants. Participants who developed type 2 diabetes were more frequently observed in the higher quintiles of both indices, with the highest concentration in Q5. This pattern was more apparent for HOMA-IR than for the TyG index. Supplementary Tables S2-S5 summarize the baseline characteristics of men and women based on the quintiles of TyG index and HOMA-IR changes. Other baseline variables, including total cholesterol and insulin levels, showed statistically significant differences across the quintiles, indicating distinct participant profiles. Notably, Q1 represented the group with the greatest decrease in the TyG index and HOMA-IR over time, and their baseline values were higher than those of the other quintiles. For example, in men, the baseline TyG index and HOMA-IR averages in Q1 were 8.84 and 1.93, respectively, which decreased to 8.28 and 1.49 in the second observation, showing a substantial reduction (Supplementary Table S2). This suggests that individuals in Q1 had higher baseline values, allowing for a greater potential reduction^22^*.*

### Association between the changes in TyG index ∙ HOMA-IR and incidence of type 2 diabetes

Tables 2 and 3 present the associations between changes in the TyG index and HOMA-IR and the risk of incident type 2 diabetes in men and women, respectively. In both sexes, higher quintiles of the TyG index change were associated with progressively higher incidence rates and risks of developing type 2 diabetes. The proportional hazards assumption was not violated for the primary exposure variables in either sex (all *P* > 0.05).

**Table 2:** Risk of incident type 2 diabetes according to quintiles of TyG Index and HOMA-IR change in men.

Quintile	Participants	Events (n)	Duration (PY)	Incidence rate (per 10^3^ PY)	Age-adjusted HR (95% CI)	Multivariable-adjusted HR (95% CI)	
						Model 1	Model 2
TyG index							
Q1 (− 2.75, − 0.28)	28,212	774	104,891.9	7.4	1.04 (0.94, 1.15)	0.70 (0.63, 0.78)	0.71 (0.64, 0.79)
Q2 (− 0.28, − 0.03)	28,212	697	107,590.5	6.5	0.92 (0.83, 1.02)	0.81 (0.73, 0.90)	0.81 (0.73, 0.90)
Q3 (− 0.03, 0.18)	28,212	770	110,682.2	7.0	Reference	Reference	Reference
Q4 (0.18, 0.43)	28,212	770	111,485.7	6.9	1.02 (0.92, 1.12)	1.20 (1.08, 1.33)	1.20 (1.08, 1.33)
Q5 (0.43, 2.82)	28,211	830	108,375.6	7.7	1.19 (1.08, 1.31)	1.72 (1.55, 1.90)	1.72 (1.55, 1.91)
				*p* for trend	0.004	< 0.001	< 0.001
HOMA-IR							
Q1 (− 8.00, − 0.50)	28,212	825	99,048.2	8.3	1.67 (1.50, 1.86)	0.71 (0.63, 0.80)	0.72 (0.64, 0.81)
Q2 (− 0.50, − 0.04)	28,212	571	108,990.2	5.2	1.02 (0.91, 1.14)	0.85 (0.76, 0.96)	0.85 (0.76, 0.96)
Q3 (− 0.04, 0.32)	28,212	578	113,308.6	5.1	Reference	Reference	Reference
Q4 (0.32, 0.82)	28,212	689	113,773.2	6.1	1.21 (1.08, 1.35)	1.17 (1.04, 1.31)	1.16 (1.03, 1.30)
Q5 (0.82, 8.64)	28,211	1,178	107,905.8	10.9	2.32 (2.10, 2.56)	1.67 (1.50, 1.85)	1.63 (1.47, 1.81)
				*p* for trend	< 0.001	< 0.001	< 0.001

CI, confidence interval; eGFR, estimated glomerular filtration rate; HOMA-IR, homeostasis model assessment of insulin resistance; HR, hazard ratio; PY, person-years; Q, quintile; TyG index, triglyceride-glucose index.

Median (IQR) follow-up duration: 3.14 (6.42) years for men. Q3 was used as the reference group for all quintile-based analyses.

Model 1 was adjusted for age, examination center, body mass index, education, smoking status, alcohol intake, regular physical activity, history of hypertension, use of lipid-lowering medication, eGFR, baseline TyG index, and baseline HOMA-IR.

Model 2 was adjusted for variables in Model 1 and total cholesterol, high-density lipoprotein cholesterol, low-density lipoprotein cholesterol, and uric acid.

**Table 3:** Risk of incident type 2 diabetes according to quintiles of TyG Index and HOMA-IR change in women.

Quintile	Participants	Events (n)	Duration (PY)	Incidence rate (per 10^3^ PY)	Age-adjusted HR (95% CI)	Multivariable-adjusted HR (95% CI)	
						Model 1	Model 2
TyG index							
Q1 (− 2.74, − 0.28)	26,107	212	98,537.9	2.2	1.16 (0.96, 1.42)	0.59 (0.47, 0.73)	0.60 (0.48, 0.76)
Q2 (− 0.28, − 0.05)	26,107	184	100,138.4	1.8	0.99 (0.80, 1.21)	0.81 (0.65, 1.02)	0.81 (0.65, 1.01)
Q3 (− 0.05, 0.15)	26,107	186	100,417.0	1.9	Reference	Reference	Reference
Q4 (0.15, 0.38)	26,106	192	101,125.3	1.9	1.04 (0.85, 1.27)	1.30 (1.04, 1.62)	1.28 (1.03, 1.60)
Q5 (0.38, 3.71)	26,106	295	100,518.2	2.9	1.68 (1.40, 2.02)	2.27 (1.85, 2.77)	2.18 (1.78, 2.67)
				*p* for trend	< 0.001	< 0.001	< 0.001
HOMA-IR							
Q1 (− 7.20, − 0.49)	26,107	249	91,109.4	2.7	2.33 (1.88, 2.88)	0.89 (0.70, 1.15)	0.92 (0.71, 1.17)
Q2 (− 0.49, − 0.09)	26,107	158	101,174.6	1.6	1.26 (1.00, 1.59)	1.13 (0.88, 1.46)	1.14 (0.89, 1.47)
Q3 (− 0.09, 0.23)	26,107	130	104,616.7	1.2	Reference	Reference	Reference
Q4 (0.23, 0.66)	26,106	153	104,396.7	1.5	1.21 (0.96, 1.53)	1.26 (0.97, 1.63)	1.23 (0.96, 1.60)
Q5 (0.66, 8.46)	26,106	379	99,439.4	3.8	3.37 (2.76, 4.12)	2.24 (1.80, 2.80)	2.14 (1.71, 2.67)
				*p* for trend	< 0.001	< 0.001	< 0.001

CI, confidence interval; eGFR, estimated glomerular filtration rate; HOMA-IR, homeostasis model assessment of insulin resistance; HR, hazard ratio; PY, person-years; Q, quintile; TyG index, triglyceride-glucose index.

Median (IQR) follow-up duration: 3.24 (6.39) years for women. Q3 was used as the reference group for all quintile-based analyses.

Model 1 was adjusted for age, examination center, body mass index, education, smoking status, alcohol intake, regular physical activity, history of hypertension, use of lipid-lowering medication, eGFR, baseline TyG index, baseline HOMA-IR, and menopause (for women).

Model 2 was adjusted for the variables in Model 1, total cholesterol, high-density lipoprotein cholesterol, low-density lipoprotein cholesterol, and uric acid.

Among men, the incidence rates ranged from 6.5 to 7.7 per 1000 person-years across the TyG index quintiles and from 5.1 to 10.9 across the HOMA-IR quintiles, showing a broader range and higher rates in the upper quintiles. In the TyG index quintiles, the fully adjusted HRs were lowest in Q1 (0.71, 95% CI 0.64–0.79) and highest in Q5 (1.72, 95% CI 1.55–1.91). A similar pattern was observed for HOMA-IR, with HRs increasing from 0.72 (Q1, 95% CI: 0.64–0.81) to 1.63 (Q5, 95% CI 1.47–1.81). All trends were statistically significant across quintiles (*p* for trend < 0.001).

Among women, the incidence rates ranged from 1.8 to 2.9 per 1000 person-years across the TyG index change quintiles and from 1.2 to 3.8 across the HOMA-IR change quintiles, respectively. In the TyG index change quintiles, the fully adjusted HRs were lowest in Q1 (0.60, 95% CI 0.48–0.76) and highest in Q5 (2.18, 95% CI 1.78–2.67). For HOMA-IR, the HRs remained relatively stable across the lower quintiles and then rose sharply in the highest quintile (HR 2.14, 95% CI 1.71–2.67), which differed from that of the TyG index. All trends were statistically significant across quintiles (*p* for trend < 0.001).

Restricted cubic spline analyses showed a progressive increase in the risk of incident type 2 diabetes with increasing TyG and HOMA-IR levels (Supplementary Figure S1). Tests for nonlinearity were statistically significant in all models (men: TyG index, *P* < 0.001; HOMA-IR, *P* = 0.014; women: TyG index, *P* = 0.001; HOMA-IR, *P* = 0.001). However, the associations were monotonic across the observed range, without evidence of abrupt threshold effects.

Although several metabolic variables differed between participants with and without extreme baseline HOMA-IR values (≥ 10), family history of diabetes did not differ according to exclusion status (Supplementary Table S8). The results were consistent across the alternative approaches for handling extreme HOMA-IR values (Supplementary Table S9). In the sensitivity analyses, baseline covariates were replaced with cardiometabolic factors measured at the second examination, including BMI, blood pressure, smoking status, alcohol intake, and the use of medications. These associations were generally consistent across the alternative model specifications (Supplementary Table S10). In sensitivity analyses, modeling changes as continuous variables (per 1–SD increase), increases in both the TyG index and HOMA-IR were significantly associated with a higher risk of type 2 diabetes in men and women (all *p* < 0.001), with results consistent across multivariable models (Supplementary Tables S11 and S12).

### Subgroup analyses

**Fig. 1:**
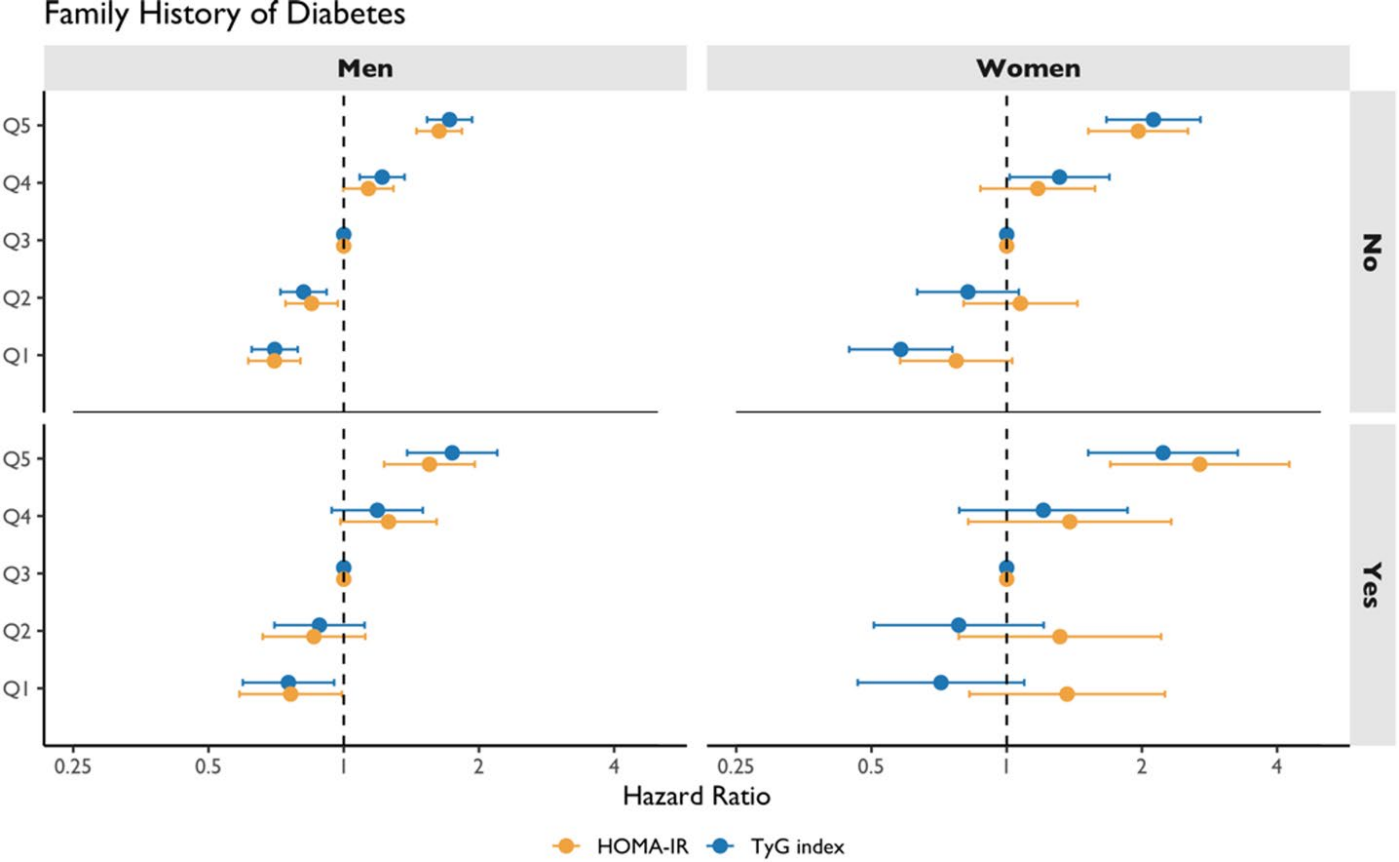
Forest plot of hazard ratios for incident type 2 diabetes according to quintiles of changes in the TyG index and HOMA-IR, stratified by sex and family history of diabetes. Hazard ratios (HRs) [95% confidence intervals (CIs)] for incident type 2 diabetes according to quintiles of change in the TyG index and HOMA-IR, stratified by sex and family history of diabetes, were estimated using a fully adjusted model (Model 2). Q3 was the reference group. No statistically significant interactions were observed between the family history of diabetes and exposure variables (all *P* > 0.7); therefore, subgroup patterns should be interpreted as exploratory.

Subgroup analyses were conducted to examine the associations between changes in the TyG index and HOMA-IR and the risk of incident type 2 diabetes across strata of family history of diabetes. The results are presented as forest plots in Fig. 1. Formal interaction tests were performed to evaluate the potential effect modification. A statistically significant interaction was observed between HOMA-IR change and sex (*P* = 0.003), whereas no significant interaction was observed between TyG index change and sex (*P* = 0.093). No significant interactions were found for family history of diabetes (all *P* > 0.7); therefore, subgroup findings stratified by family history should be interpreted as exploratory findings.

In men, both the TyG index and HOMA-IR changes showed a generally monotonic increase in HRs across quintiles, with broadly similar patterns observed regardless of the family history status (Supplementary Table S6). In women without a family history of diabetes, both the TyG index and HOMA-IR showed progressive increases in the risk of type 2 diabetes across quintiles, with a relatively more pronounced gradient observed for the TyG index than for HOMA-IR. In contrast, among women with a family history of diabetes, the TyG index change maintained a positive gradient across quintiles, whereas the HOMA-IR change showed attenuated and non-significant associations with wide confidence intervals overlapping the null hypothesis. These patterns are hypothesis-generating and warrant confirmation in future studies (Supplementary Table S7).

### Sensitivity analyses

Sensitivity analyses using multiple imputations and inverse probability weighting yielded results consistent with those of the primary complete-case analysis (Supplementary Table S13). To further address the potential influence of regression to the mean, we conducted additional sensitivity analyses using residual change scores and joint modeling of baseline and follow-up values. Results were consistent in direction and dose–response pattern with the primary analysis across sex and index (Supplementary Table S14).

## Discussion

In this large cohort of generally healthy young and middle-aged adults, serial changes in both TyG and HOMA-IR were significantly associated with the risk of developing type 2 diabetes in a sex-stratified manner. Increases in both indices were associated with a higher risk of diabetes, whereas decreases were associated with a lower risk, with associations generally following a monotonic pattern across quintiles. Notably, the TyG index showed more consistent associations across quintiles and analytical models than HOMA-IR, whereas the associations for HOMA-IR changes were more variable, particularly in the lower quintiles. These findings suggest that longitudinal changes in the TyG index may provide more stable predictive information for type 2 diabetes risk than HOMA-IR.

Previous studies have demonstrated that the TyG index predicts incident diabetes comparably to or better than HOMA-IR, largely based on cross-sectional or baseline measurements^23–25^. Our study extends this evidence by focusing on temporal changes in these indices and capturing the dynamic shifts in metabolic status over time. To our knowledge, this study is the first large-scale comparison of temporal changes in both indices in a cohort of young and generally healthy adults. Although both the TyG index and HOMA-IR are considered surrogate markers of insulin resistance, their biological underpinnings differ in ways that may influence their performance over time. The TyG index integrates fasting triglyceride and glucose levels, thereby capturing the combined hepatic and peripheral insulin resistance^26,27^. Elevated triglyceride levels reflect impaired suppression of hepatic lipolysis and increased free fatty acid flux, which in turn promotes ectopic lipid accumulation and further impairs insulin signaling—a pathway closely linked to the pathogenesis of type 2 diabetes^23,28,29^. In contrast, HOMA-IR is derived from fasting insulin and glucose concentrations and therefore primarily reflects hepatic insulin resistance and residual beta cell function^30^. Fasting insulin levels are influenced not only by insulin resistance but also by insulin secretory capacity, which may vary considerably across individuals and over time, potentially introducing biological noise into longitudinal assessments^31–34^. These biological differences may contribute to the more variable associations observed for HOMA-IR changes in our study.

Several methodological factors may also contribute to the observed differences between the TyG index and HOMA-IR change associations. HOMA-IR relies on fasting insulin measurements, which are subject to greater biological fluctuations and inter-assay variability than the lipid and glucose components of the TyG index^35,36^. This inherent measurement variability in insulin assays may attenuate the observed associations between HOMA-IR changes and diabetes risk, particularly in longitudinal settings where repeated measurements are required. In contrast, the TyG index, derived solely from fasting triglycerides and glucose, may provide more stable repeated measurements over time^37^. Therefore, the more variable associations observed for HOMA-IR may partly reflect measurement-related factors rather than true biological differences in insulin resistance trajectory. Although increases in HOMA-IR were associated with a higher risk of type 2 diabetes, reductions in HOMA-IR over time showed less consistent associations, which may reflect regression to the mean or underlying variability in insulin measurements^35,36^. These considerations should be taken into account when interpreting the comparative findings of this study.

The present findings underscore the clinical value of monitoring longitudinal changes in metabolic indices rather than relying solely on single time-point measurements. Previous studies on the TyG index and HOMA-IR have predominantly focused on single-point assessments^20,21^, leaving the incremental predictive value of longitudinal TyG index and HOMA-IR changes for incident type 2 diabetes largely unexamined, particularly in large-scale cohorts of young and middle-aged adults. The key conceptual premise of our study is that the trajectory of a metabolic marker carries prognostic information beyond its absolute level^38–40^. Individuals whose TyG index or HOMA-IR progressively increases, even within the conventionally normal range, may face an accumulating metabolic burden that is not captured by a static snapshot. Conversely, those who demonstrate improvement from an initially unfavorable level may experience a meaningful reduction in the risk of diabetes. Notably, individuals in the highest quintile of TyG index or HOMA-IR change demonstrated markedly elevated diabetes risk, whereas those in the lowest quintile, reflecting the greatest improvement, showed significantly reduced risk, underscoring the bidirectional clinical relevance of metabolic trajectory monitoring. To better capture the bidirectional clinical implications of metabolic trajectory, the third quintile—representing a relatively stable trajectory—was used as the reference group rather than the conventional first quintile. Under this framing, hazard ratios below 1.0 reflect reduced risk associated with metabolic improvement, whereas those above 1.0 reflect increased risk associated with worsening, both relative to a stable trajectory. This principle is consistent with evidence from other metabolic markers^38^, where longitudinal changes in metabolic health status^40,41^, non-HDL to HDL cholesterol ratio^39^, and natriuretic peptides^42^ have been shown to provide incremental predictive value for cardiometabolic outcomes beyond baseline levels.

Subgroup analyses suggested that the associations between changes in both the TyG index and HOMA-IR with diabetes risk were broadly consistent across the strata. Variations observed by sex and family history of diabetes should be interpreted cautiously, as no statistically significant interactions were observed for family history of diabetes (all *P* > 0.7); therefore, these findings should be interpreted as exploratory and hypothesis-generating. These descriptive patterns may reflect differences in how each index captures insulin resistance, with the TyG index potentially being less influenced by variability in insulin secretion than the HOMA-IR^43–45^, though these proposed mechanisms remain speculative and require confirmation in future studies.

These findings have several clinical implications. As the TyG index can be calculated from routinely measured fasting glucose and triglyceride levels, it offers a practical means of assessing metabolic trajectories without the need for insulin assays, which may be of value in settings where insulin measurements are unavailable. These observations may be particularly relevant in younger populations in whom overt hyperglycemia has not yet developed, although prospective validation in diverse settings is warranted before broader clinical application. Individuals whose TyG index or HOMA-IR shows a consistent upward trajectory across repeated health examinations may represent a higher-risk group and may help identify individuals who could benefit from earlier preventive strategies, such as lifestyle modification counseling, even in the absence of overt dysglycemia. Whether serial monitoring of these indices provides incremental predictive value beyond established risk factors—such as fasting glucose, BMI, or family history—remains to be formally evaluated. However, longitudinal changes in these indices may help capture early metabolic deterioration or improvement that is not fully reflected by single time-point measurements. Given that these indices are derived from routinely collected laboratory values in periodic health examinations, their integration into existing screening frameworks would not require additional testing and may therefore represent a pragmatically low-cost enhancement to conventional risk assessment, particularly in settings such as national health screening programs where repeated examinations are routinely conducted^46^. Conversely, individuals demonstrating improvement in these indices may reflect favorable metabolic changes and may benefit from continued reinforcement of health-promoting behaviors, as such trajectories are associated with a lower future risk of diabetes. The strengths of this study include its exceptionally large sample size, repeated metabolic measurements, and focus on a relatively young and metabolically healthy population, enabling the evaluation of temporal changes in insulin resistance indices before the onset of diabetes mellitus. The inclusion of both the TyG index and HOMA-IR enabled a direct comparison between lipid, glucose, and insulin resistance markers.

This study had several limitations. First, given the observational nature of this study, causal relationships between changes in insulin resistance indices and diabetes risk cannot be firmly established. Several alternative explanations merit consideration. Changes in metabolic indices may partly reflect concurrent unmeasured lifestyle modifications between examinations. Regression to the mean may also contribute to apparent improvements in individuals with extreme baseline values. To address this concern, sensitivity analyses using residual change scores and joint modeling of baseline and follow-up values yielded results consistent with the primary analysis, suggesting that regression to the mean is unlikely to fully explain our findings; nevertheless, residual bias inherent to observational studies of change cannot be entirely excluded. Furthermore, as this was an observational study, residual confounding cannot be excluded. Although several lifestyle and behavioral factors were included as covariates, including smoking status, alcohol intake, regular physical activity, and education level, dietary habits and socioeconomic status were not captured in our dataset and, therefore, could not be adjusted for in the analyses, and their potential influence on both changes in metabolic indices and diabetes risk cannot be ruled out. Unhealthy dietary patterns, such as high intake of refined carbohydrates and saturated fats, and lower socioeconomic status are associated with both worsening metabolic indices and higher diabetes risk, suggesting that residual confounding from these factors could potentially bias the observed associations away from the null (i.e., toward stronger associations). However, given that our study population consisted predominantly of employed, health-screened adults with relatively homogeneous socioeconomic backgrounds, the variability in unmeasured dietary and socioeconomic factors may be reduced compared with the general population, which could limit the magnitude of such residual confounding. Second, insulin concentrations were measured at only two time points, which may have introduced measurement variability and limited the precision of HOMA-IR estimation. Extremely high HOMA-IR values may represent severe insulin resistance or reflect variability in fasting insulin measurements, which may complicate their interpretation in longitudinal studies. Although these extreme values were excluded from the primary analysis, sensitivity analyses using alternative approaches, including analyses with all values retained and winsorization, yielded consistent results, supporting the robustness of our findings. Third, the TyG index and HOMA-IR are surrogate markers and may not fully capture the complex aspects of insulin resistance, such as postprandial dynamics and tissue-specific differences in insulin resistance. Fourth, our study population consisted predominantly of young, health-screened Korean adults employed in occupational settings, and the generalizability of our findings to other ethnic groups or higher-risk populations should be interpreted with caution when extrapolating these findings to broader populations. In particular, populations with higher background diabetes prevalence, different body composition profiles, or limited access to routine health screening may exhibit different absolute risk thresholds and effect sizes for TyG index and HOMA-IR changes. Furthermore, the health screening context of our cohort may not be representative of the general population or primary care settings in other countries. Future studies involving diverse ethnic groups and healthcare settings are warranted to validate these findings. In addition, because the primary exposures were defined as changes between the first and second examinations, baseline covariates were used in the main models to maintain temporal ordering. Although residual or time-varying confounding factors cannot be entirely excluded, additional adjustment for second examination covariates did not materially alter these associations. Furthermore, although selection bias related to the requirement of two examinations and complete covariate data is possible, sensitivity analyses using multiple imputations and inverse probability weighting yielded consistent results, reinforcing the robustness of our findings.

In conclusion, in this large longitudinal cohort of young and middle-aged adults, increases in both the TyG index and HOMA-IR were significantly associated with incident type 2 diabetes in a sex-stratified analysis. The TyG index showed a generally consistent association across subgroups, whereas HOMA-IR showed more variable patterns across the analytical models. These findings suggest that monitoring longitudinal changes in these metabolic indices may provide incremental information for diabetes risk assessment; however, further studies in diverse populations are needed to confirm these observations.

## Materials and methods

### Study population

The Kangbuk Samsung Health Study (KSHS) is a retrospective cohort study of adults who underwent regular health screenings at the Total Healthcare Centers of Kangbuk Samsung Hospital in Seoul and Suwon, Republic of Korea. The examinations included standardized questionnaires and laboratory assessments^47^.

**Fig. 2:**
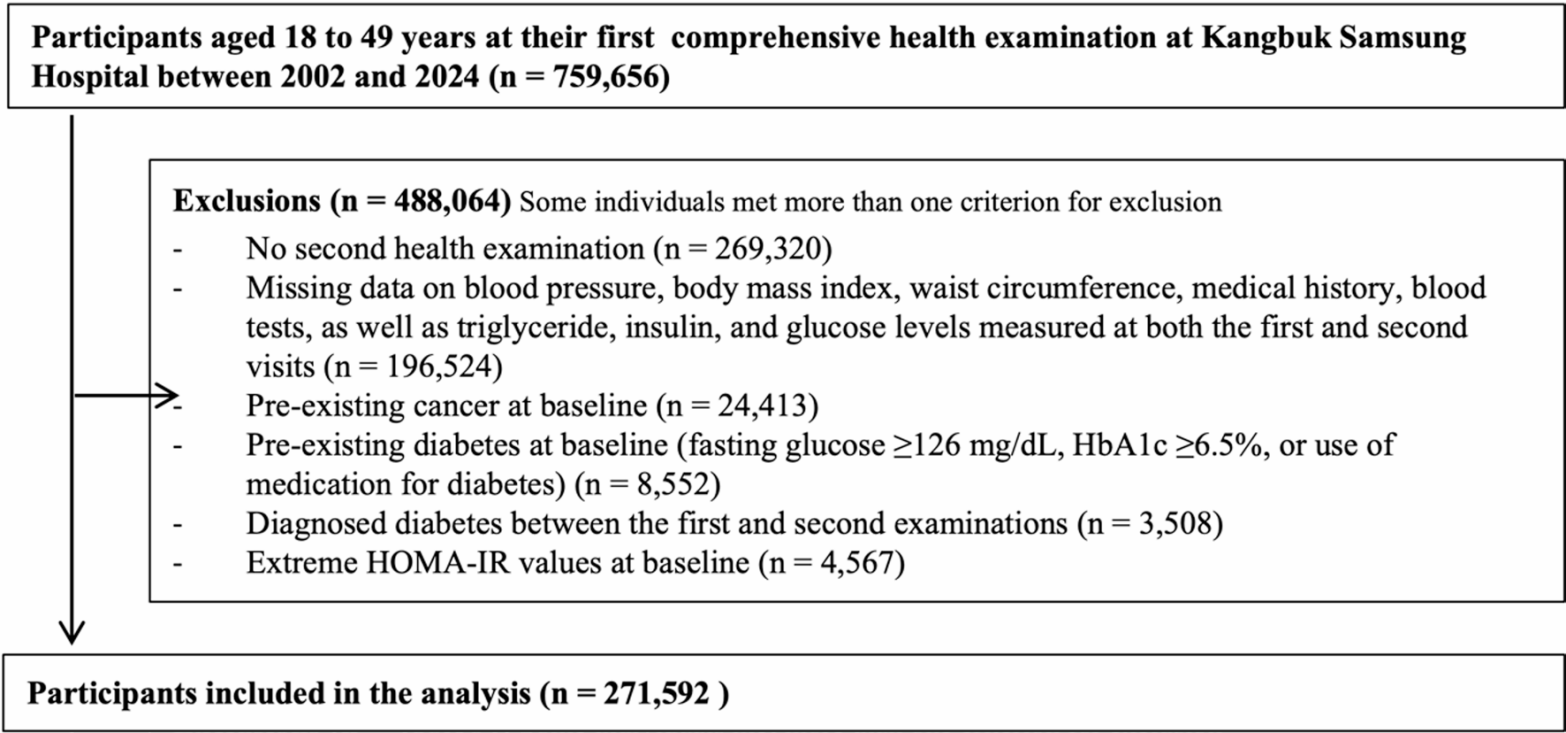
Flowchart of participants.

This study initially included individuals aged 18–49 years who underwent health examinations between 2002 and 2024 (n = 759,656 individuals). Participants who did not undergo a second health examination were excluded. We further excluded participants with missing key covariates at either visit, those with pre-existing diabetes at the first examination, those who developed diabetes between the first and second examinations, those with a history of cancer prior to baseline^22,33,48^, and those with extreme baseline HOMA-IR values (≥ 10), corresponding to approximately the upper 1% of the distribution of HOMA-IR values. The final cohort comprised 271,592 participants (Fig. 2).

### Definitions of exposure and study outcome

Exposure was defined as a change in the TyG index and HOMA-IR between the first and second health examinations. The TyG index for each assessment was computed as ln[triglyceride(mg/dL) × glucose (mg/dL)/2], following established methods^57^, and HOMA-IR was calculated as follows: insulin(µU/mL) × fasting blood glucose (mg/dL)/405. Participants with HOMA-IR values ≥ 10 were excluded from the primary analysis (n = 4,567). These values corresponded to approximately the upper 1% of the distribution and were substantially higher than those typically observed in the general population, suggesting either severe insulin resistance or potential measurement variability related to fasting insulin concentrations^49,50^. Because HOMA-IR is directly derived from fasting insulin levels, it is particularly sensitive to short-term biological variations and assay-related errors, which may disproportionately affect estimates of longitudinal changes. Sensitivity analyses using alternative approaches, including analyses without exclusion and winsorization, yielded similar results.

The remaining participants were categorized into quintiles for each index to reflect the distribution and variability within the population, similar to previous studies^2,51,52^. The third quintile (Q3), representing participants with minimal net change between examinations (i.e., a stable trajectory), was prespecified as the reference category. This enabled comparison of participants with metabolic improvement (Q1–Q2) or deterioration (Q4–Q5) relative to a stable group, consistent with prior longitudinal analyses of metabolic change conducted within the same cohort^8,53^.

The quintiles of change in the triglyceride–glucose (TyG) index were defined separately for men and women based on the distribution of change values in the study population. In men, the cut-off values were − 2.75 to − 0.28 (Q1), − 0.28 to − 0.03 (Q2), − 0.03 to 0.18 (Q3), 0.18 to 0.43 (Q4), and 0.43 to 2.82 (Q5). In women, the corresponding ranges were − 2.74 to − 0.28 (Q1), − 0.28 to − 0.05 (Q2), − 0.05 to 0.15 (Q3), 0.15 to 0.38 (Q4), and 0.38 to 3.71 (Q5). Similarly, quintiles of change in HOMA-IR were defined separately by sex based on the distribution of change values. In men, the cut-off values were − 8.00 to − 0.50 (Q1), − 0.50 to − 0.04 (Q2), − 0.04 to 0.32 (Q3), 0.32 to 0.82 (Q4), and 0.82 to 8.64 (Q5), whereas in women they were − 7.20 to − 0.49 (Q1), − 0.49 to − 0.09 (Q2), − 0.09 to 0.23 (Q3), 0.23 to 0.66 (Q4), and 0.66 to 8.46 (Q5).

The primary outcome of this study was incident type 2 diabetes, defined as FBG ≥ 126 mg/dL, glycated hemoglobin (HbA1c) ≥ 6.5%, or use of medication for diabetes mellitus after the baseline visit^54,55^. Follow-up was initiated at the second examination and continued until the participants developed type 2 diabetes, reached their final examination date, or December 31, 2024, whichever occurred first. While the exposure of interest was specified as the change between the first and second examinations, all other covariates for adjustment were measured at the initial visit and treated as baseline characteristics. Because the primary exposures were defined as changes between the first and second examinations, baseline covariates were used in the main models to preserve the temporal ordering and reduce the risk of over-adjustment. This design enabled the assessment of the association between changes in the TyG index and HOMA-IR and the subsequent risk of T2D, while accounting for confounders present prior to the exposure period.

### Measurements

Demographic factors, including age, sex, and educational level, as well as health-related behaviors, medical history, and medication use, were recorded during health checkups using self-reported questionnaires. Smoking status was classified into three categories: never, former, and current smokers. Alcohol intake was divided into < 20 or ≥ 20 g/day for men and < 10 or ≥ 10 g/day for women. Regular physical activity was defined as vigorous physical activity at least three times per week.

Body measurements were taken by trained nurses who recorded the height and weight of the participants while they were wearing light clothes. BMI was calculated as weight in kilograms divided by height in meters squared (kg/m^2^). Waist circumference was measured by two trained staff members to the nearest 0.1 cm while the participants stood with their weight evenly on both feet, arms at their sides, and facing the front. Blood tests were performed using an automated device after 10 min of rest period. Blood samples were obtained after a 10-h fast and included measurements of total cholesterol, triglyceride, FBG, insulin, HbA1c, and uric acid levels.

A family history of diabetes was defined based on self-reported information indicating whether any relatives had been diagnosed with diabetes by a health care professional.

### Statistical analyses

For variable characterization, continuous baseline measures are presented as mean ± standard deviation, and categorical variables are presented as frequencies and percentages. The analyses were stratified by sex, with male and female participants being assessed independently. Group differences across the quintiles of TyG index and HOMA-IR changes were evaluated using one-way analysis of variance (ANOVA) for continuous variables and chi-square tests for categorical variables.

Hazard ratios (HRs) and 95% confidence intervals (CIs) for incident type 2 diabetes were estimated using Cox proportional hazards models across the TyG index and HOMA-IR quintiles. To ensure a clear temporal sequence between exposure assessment and outcome occurrence, the follow-up time was defined from the date of the second health examination, at which changes in the TyG index and HOMA-IR were fully ascertained. Person-years were calculated by summing the individual follow-up times for all participants. Three modeling approaches were applied: (1) an age-adjusted model; (2) multivariate model 1, adjusted for age, study center (Seoul or Suwon), body mass index (BMI), education level, smoking status, alcohol intake, regular physical activity, diabetes history, hypertension history, use of lipid-lowering medications, estimated glomerular filtration rate (eGFR), TyG index, HOMA-IR at baseline, and menopausal status for women; and (3) multivariate model 2, additionally adjusted for total cholesterol, high-density lipoprotein cholesterol (HDL-C), low-density lipoprotein cholesterol (LDL-C), and uric acid levels. The interval between the first and second examinations reflected the exposure assessment window, which had a mean of 2.11 years (SD, 1.30); therefore, it was not included as a covariate in the primary Cox proportional hazards models to avoid potential overadjustment^22^. Including baseline values as covariates allows the change score to reflect variation independent of the initial level and helps mitigate regression-to-the-mean effects^56^. Multicollinearity between baseline values and change scores was assessed using generalized variance inflation factors (GVIFs), with no evidence of substantial collinearity (all ${GVIF}^{1/(2\cdot \mathrm{D}\mathrm{f})}<2.0$).

Subgroup analyses were conducted according to the presence or absence of a family history of diabetes. The proportional hazards assumption was assessed for all model covariates using Schoenfeld residuals, and no violations were detected for the primary exposure variables. Restricted cubic spline functions with knots placed at the 5th, 35th, 65th, and 95th percentiles were incorporated into the Cox proportional hazards models to evaluate potential nonlinear dose–response relationships.

Subgroup analyses were performed using forest plot analysis. These plots display HRs and 95% CIs for each quintile within subgroups, with point estimates shown as squares and CIs as horizontal lines. A vertical reference line at HR = 1.0, which corresponds to Q3 in this study, indicates no effect. Forest plots allow for a quick visual comparison of the consistency or heterogeneity of the association between TyG index or HOMA-IR changes and type 2 diabetes risk across different baseline characteristics. For model covariates not specified as exclusion criteria, missing values were handled through complete case analysis, with participants excluded list-wise if any covariate value was absent. To assess potential bias due to missing covariate data, multiple imputations using chained equations were performed, including exposures, outcomes, and all covariates in the fully adjusted model. Inverse probability weighting was applied to account for potential selection bias related to the requirement of at least two health check-ups. To address potential regression to the mean, two additional sensitivity analyses were performed: (1) a residual change approach, in which residuals from regressing follow-up values on baseline values were used as the exposure, and (2) joint modeling, in which baseline and follow-up values were simultaneously included as separate covariates in the Cox model.

Statistical significance was determined using a two-sided *p*-value < 0.05, and all analyses were performed using R software version 4.4.3 (R Core Team, Vienna, Austria) with the *survival* package.

## Supplementary Information

Below is the link to the electronic supplementary material.

Supplementary Material 1

## Data Availability

The datasets generated and/or analyzed in the current study are not publicly available owing to institutional and privacy restrictions but are available from the corresponding author upon reasonable requests. The R scripts used for data management and all statistical analyses are archived at https://github.com/Sojinnn-Kim/TyG_HOMAIR_Change_T2DM.
