# Supplementary material for: Sex-stratified associations of TyG index and HOMA-IR changes with incident type 2 diabetes
Source: Sci Rep. 2026 May 8;16:21114. doi: 10.1038/s41598-026-52072-y (PMC13342541; doi:10.1038/s41598-026-52072-y)
Supplement: Supplementary file 1 — Supplementary Material 1 [file 41598_2026_52072_MOESM1_ESM.docx]

**Supplementary Information**

**Supplementary Table S1. Quintile distribution of TyG index and HOMA-IR by sex and T2DM status**

| **Variables** | **Men** | | | **Women** | | |
| --- | --- | --- | --- | --- | --- | --- |
|  | Total | T2DM | Non-T2DM | Total | T2DM | Non-T2DM |
| TyG Index |  |  |  |  |  |  |
| Q1 | 28,212 | 774 (20.15) | 27,438 (20) | 26,107 | 212 (19.83) | 25,895 (20) |
| Q2 | 28,212 | 697 (18.15) | 27,515 (20.05) | 26,107 | 184 (17.21) | 25,923 (20.02) |
| Q3 | 28,212 | 770 (20.05) | 27,442 (20) | 26,107 | 186 (17.4) | 25,921 (20.02) |
| Q4 | 28,212 | 770 (20.05) | 27,442 (20) | 26,106 | 192 (17.96) | 25,914 (20.02) |
| Q5 | 28,211 | 830 (21.61) | 27,381 (19.95) | 26,106 | 295 (27.6) | 25,811 (19.94) |
| HOMA-IR |  |  |  |  |  |  |
| Q1 | 28,212 | 825 (21.48) | 27,387 (19.96) | 26,107 | 249 (23.29) | 25,858 (19.97) |
| Q2 | 28,212 | 571 (14.87) | 27,641 (20.14) | 26,107 | 158 (14.78) | 25,949 (20.04) |
| Q3 | 28,212 | 578 (15.05) | 27,634 (20.14) | 26,107 | 130 (12.16) | 25,977 (20.07) |
| Q4 | 28,212 | 689 (17.94) | 27,523 (20.06) | 26,106 | 153 (14.31) | 25,953 (20.05) |
| Q5 | 28,211 | 1,178 (30.67) | 27,033 (19.7) | 26,106 | 379 (35.45) | 25,727 (19.87) |

**Abbreviations**: HOMA-IR, homeostasis model assessment of insulin resistance; T2DM, type 2 diabetes mellitus; TyG index, triglyceride-glucose index; Q, quintile.

Data are expressed as a number (percentage).

TyG index quintile cut-off values are, in men: Q1 (−2.75, −0.28), Q2 (−0.28, −0.03), Q3 (−0.03, 0.18), Q4 (0.18, 0.43), Q5 (0.43, 2.82); in women: Q1 (−2.74, −0.28), Q2 (−0.28, −0.05), Q3 (−0.05, 0.15), Q4 (0.15, 0.38), Q5 (0.38, 3.71).

HOMA-IR quintile cut-off values are, in men: Q1 (−8.00, −0.50), Q2 (−0.50, −0.04), Q3 (−0.04, 0.32), Q4 (0.32, 0.82), Q5 (0.82, 8.64); in women: Q1 (−7.20, −0.49), Q2 (−0.49, −0.09), Q3 (−0.09, 0.23), Q4 (0.23, 0.66), Q5 (0.66, 8.46).

**Supplementary Table S2. Baseline Characteristics of Men by Quintiles of TyG index Change**

|  | Total | TyG index change quintiles | | | | | |
| --- | --- | --- | --- | --- | --- | --- | --- |
|  |  | Q1  (-2.75, -0.28) | Q2  (-0.28, -0.03) | Q3 (-0.03, 0.18) | Q4  (0.18, 0.43) | Q5  (0.43, 2.82) | *p*-value |
| N | 141,059 | 28,212 | 28,212 | 28,212 | 28,212 | 28,211 |  |
| Age, years | 34.11±5.57 | 34.53±5.61 | 34.37±5.62 | 34.16±5.54 | 33.99±5.53 | 33.51±5.51 | <0.001 |
| Alcohol intake$\geq$20g/day (%) | 34,263 (24.94) | 7,350 (26.05) | 6,565 (23.27) | 6,612 (23.44) | 6,549 (23.21) | 7,187 (25.48) | <0.001 |
| Regular physical activity (%) | 21,519 (15.35) | 3,989 (14.14) | 3,962 (14.04) | 4,001 (14.18) | 4,277 (15.16) | 5,290 (18.75) | <0.001 |
| Smoking status (%) |  |  |  |  |  |  | <0.001 |
| Never | 52,289 (37.52) | 10,220 (36.23) | 10,418 (36.93) | 10,390 (36.83) | 10,612 (37.62) | 10,649 (37.75) |  |
| Former | 43,687 (31.35) | 8,967 (31.78) | 8,677 (30.76) | 8,672 (30.74) | 8,690 (30.8) | 8,681 (30.77) |  |
| Current | 43,375 (31.13) | 8,673 (30.74) | 8,751 (31.02) | 8,785 (31.14) | 8,591 (30.45) | 8,575 (30.4) |  |
| Education: University or more (%) | 107,678 (78.14) | 21,615 (76.62) | 22,000 (77.98) | 21,881 (77.56) | 21,739 (77.06) | 20,443 (72.46) | <0.001 |
| BMI, kg/m^2^ | 24.63±3.05 | 24.95±3.08 | 24.7±3.13 | 24.64±3.11 | 24.52±3.01 | 24.34±2.91 | <0.001 |
| Waist circumference, cm | 85.72±8.2 | 86.52±8.22 | 85.9±8.38 | 85.83±8.33 | 85.45±8.13 | 84.91±7.85 | <0.001 |
| SBP, mmHg | 113.93±10.87 | 115.1±11.11 | 114.13±10.94 | 113.76±10.85 | 113.41±10.6 | 113.25±10.72 | <0.001 |
| DBP, mmHg | 72.02±8.87 | 73.21±9.11 | 72.31±9 | 71.99±8.86 | 71.56±8.64 | 71.04±8.6 | <0.001 |
| Total cholesterol, mg/dL | 195.92±33.88 | 199.72±35 | 196.38±33.36 | 195.34±33.34 | 194.62±33.62 | 193.53±33.75 | <0.001 |
| HbA1c, % | 5.45±0.25 | 5.46±0.26 | 5.45±0.26 | 5.45±0.25 | 5.44±0.25 | 5.43±0.25 | <0.001 |
| Insulin, uIU/mL | 6.93±4.09 | 8.02±4.44 | 7.38±4.24 | 7.01±4.06 | 6.52±3.8 | 5.69±3.46 | <0.001 |
| eGFR, mL/min/1.73m^2^ | 102.19±12.83 | 102.63±12.74 | 102.22±12.78 | 102.34±12.77 | 101.9±12.85 | 101.86±12.99 | <0.001 |
| Uric acid, mg/dL | 6.32±1.23 | 6.32±1.25 | 6.27±1.22 | 6.3±1.22 | 6.32±1.23 | 6.41±1.24 | <0.001 |
| FBG, mg/dL | 93.85±7.96 | 96.19±7.61 | 94.91±7.58 | 94.09±7.62 | 93.06±7.66 | 91.02±8.35 | <0.001 |
| Triglycerides, mg/dL | 125.25±83.43 | 166.59±115.35 | 133.26±76.07 | 122.5±70.65 | 110.12±67.34 | 93.77±56.46 | <0.001 |
| TyG index |  |  |  |  |  |  |  |
| 1^st^ | 8.52±0.56 | 8.84±0.54 | 8.62±0.51 | 8.53±0.51 | 8.41±0.51 | 8.22±0.53 | <0.001 |
| 2^nd^ | 8.6±0.56 | 8.28±0.53 | 8.47±0.51 | 8.6±0.51 | 8.71±0.51 | 8.93±0.54 | <0.001 |
| HOMA-IR |  |  |  |  |  |  |  |
| 1^st^ | 1.63±1.03 | 1.93±1.13 | 1.75±1.07 | 1.66±1.02 | 1.52±0.95 | 1.31±0.86 | <0.001 |
| 2^nd^ | 1.81±1.14 | 1.49±1.02 | 1.71±1.1 | 1.84±1.14 | 1.92±1.15 | 2.09±1.2 | <0.001 |
| History of hypertension (%) | 10,234 (7.26) | 2,296 (8.14) | 2,067 (7.33) | 2,011 (7.13) | 1,933 (6.85) | 1,927 (6.83) | <0.001 |
| Usage of lipid-lowering medication (%) | 2,098 (1.49) | 437 (1.55) | 405 (1.44) | 410 (1.45) | 423 (1.5) | 423 (1.5) | 0.822 |
| Family history of diabetes (%) | 15,534 (11.01) | 3,195 (11.32) | 3,105 (11.01) | 3,116 (11.04) | 3,022 (10.71) | 3,096 (10.97) | 0.241 |

**Abbreviations**: BMI, body mass index; ; DBP, diastolic blood pressure; eGFR, estimated Glomerular Filtration Rate, FBG, fasting blood glucose; HbA1c, glycated hemoglobin; HOMA-IR, homeostasis model assessment of insulin resistance; SBP, systolic blood pressure; TyG index, triglyceride-glucose index; Q, quintile.

Data are expressed as mean$\pm$standard deviation or a number (percentage). One-way ANOVA was used for continuous variables and chi-square test for categorical variables.

Alcohol intake was defined as ≥20 g/day for men and ≥10 g/day for women. Regular physical activity defined as vigorous exercise frequency $\geq$3times/week.

**Supplementary Table S3. Baseline Characteristics of Men by Quintiles of HOMA-IR Change**

|  | Total | HOMA-IR change quintiles | | | | | |
| --- | --- | --- | --- | --- | --- | --- | --- |
|  |  | Q1  (-8.00, -0.50) | Q2  (-0.50, -0.04) | Q3 (-0.04, 0.32) | Q4  (0.32, 0.82) | Q5  (0.82, 8.64) | *p*-value |
| N | 141,059 | 28,212 | 28,212 | 28,212 | 28,212 | 28,211 |  |
| Age, years | 34.11±5.57 | 34.14±5.6 | 34.33±5.63 | 34.31±5.62 | 34.12±5.56 | 33.66±5.43 | <0.001 |
| Alcohol intake$\geq$20g/day (%) | 34,263 (24.94) | 6,666 (23.63) | 6,841 (24.25) | 6,832 (24.22) | 6,841 (24.25) | 7,083 (25.11) | 0.002 |
| Regular physical activity (%) | 21,519 (15.35) | 3,637 (12.89) | 4,423 (15.68) | 4,547 (16.12) | 4,546 (16.11) | 4,366 (15.48) | <0.001 |
| Smoking status (%) |  |  |  |  |  |  | <0.001 |
| Never | 52,289 (37.52) | 10,742 (38.08) | 10,487 (37.17) | 10,246 (36.32) | 10,411 (36.9) | 10,403 (36.88) |  |
| Former | 43,687 (31.35) | 8,915 (31.6) | 8,677 (30.76) | 8,833 (31.31) | 8,639 (30.62) | 8,623 (30.57) |  |
| Current | 43,375 (31.13) | 8,240 (29.21) | 8,700 (30.84) | 8,755 (31.03) | 8,792 (31.16) | 8,888 (31.51) |  |
| Education: University or more (%) | 107,678 (78.14) | 21,391 (75.82) | 21,818 (77.34) | 21,922 (77.7) | 21,718 (76.98) | 20,829 (73.83) | <0.001 |
| BMI, kg/m^2^ | 24.63±3.05 | 25.37±3.21 | 24.2±2.85 | 23.95±2.82 | 24.24±2.85 | 25.39±3.19 | <0.001 |
| Waist circumference, cm | 85.72±8.2 | 87.71±8.4 | 84.47±7.78 | 83.85±7.74 | 84.73±7.81 | 87.84±8.36 | <0.001 |
| SBP, mmHg | 113.93±10.87 | 115.67±10.9 | 113.36±10.62 | 112.74±10.6 | 113.03±10.73 | 114.86±11.18 | <0.001 |
| DBP, mmHg | 72.02±8.87 | 73.49±9 | 71.8±8.74 | 71.22±8.63 | 71.22±8.73 | 72.38±9.06 | <0.001 |
| Total cholesterol, mg/dL | 195.92±33.88 | 198.57±34.52 | 194.41±33.14 | 193.33±32.65 | 194.96±33.39 | 198.32±35.32 | <0.001 |
| HbA1c, % | 5.45±0.25 | 5.47±0.26 | 5.43±0.25 | 5.43±0.25 | 5.44±0.25 | 5.46±0.26 | <0.001 |
| Insulin, uIU/mL | 6.93±4.09 | 10.61±4.87 | 6.57±3.05 | 5.53±2.98 | 5.42±3.11 | 6.49±3.74 | <0.001 |
| eGFR, mL/min/1.73m^2^ | 102.19±12.83 | 103.07±12.85 | 102.15±12.79 | 101.85±12.72 | 101.73±12.71 | 102.16±13.02 | <0.001 |
| Uric acid, mg/dL | 6.32±1.23 | 6.38±1.27 | 6.19±1.2 | 6.19±1.18 | 6.29±1.2 | 6.57±1.27 | <0.001 |
| FBG, mg/dL | 93.85±7.96 | 97.88±7.59 | 94.43±7.23 | 92.79±7.52 | 91.98±7.83 | 92.19±8.09 | <0.001 |
| Triglycerides, mg/dL | 125.25±83.43 | 148.41±97.96 | 121.28±77.99 | 113.1±69 | 114.91±76.56 | 128.53±87.68 | <0.001 |
| TyG index |  |  |  |  |  |  |  |
| 1^st^ | 8.52±0.56 | 8.74±0.54 | 8.51±0.53 | 8.43±0.53 | 8.42±0.55 | 8.53±0.58 | <0.001 |
| 2^nd^ | 8.6±0.56 | 8.5±0.57 | 8.46±0.55 | 8.51±0.52 | 8.64±0.53 | 8.88±0.54 | <0.001 |
| HOMA-IR |  |  |  |  |  |  |  |
| 1^st^ | 1.63±1.03 | 2.58±1.25 | 1.54±0.76 | 1.29±0.74 | 1.25±0.77 | 1.51±0.93 | <0.001 |
| 2^nd^ | 1.81±1.14 | 1.46±0.9 | 1.29±0.75 | 1.42±0.74 | 1.8±0.78 | 3.06±1.37 | <0.001 |
| History of hypertension (%) | 10,234 (7.26) | 2,464 (8.73) | 1,764 (6.25) | 1,650 (5.85) | 1,846 (6.54) | 2,510 (8.9) | <0.001 |
| Usage of lipid-lowering medication (%) | 2,098 (1.49) | 528 (1.87) | 358 (1.27) | 335 (1.19) | 335 (1.19) | 542 (1.92) | <0.001 |
| Family history of diabetes (%) | 15,534 (11.01) | 3,139 (11.13) | 3,053 (10.82) | 2,971 (10.53) | 3,067 (10.87) | 3,304 (11.71) | <0.001 |

**Abbreviations**: BMI, body mass index; ; DBP, diastolic blood pressure; eGFR, estimated Glomerular Filtration Rate, FBG, fasting blood glucose; HbA1c, glycated hemoglobin; HOMA-IR, homeostasis model assessment of insulin resistance; SBP, systolic blood pressure; TyG index, triglyceride-glucose index; Q, quintile.

Data are expressed as mean$\pm$standard deviation or a number (percentage). One-way ANOVA was used for continuous variables and chi-square test for categorical variables.

Alcohol intake was defined as ≥20 g/day for men and ≥10 g/day for women. Regular physical activity defined as vigorous exercise frequency $\geq$3times/week.

**Supplementary Table S4. Baseline Characteristics of Women by Quintiles of TyG index Change**

|  | Total | TyG index change quintiles | | | | | |
| --- | --- | --- | --- | --- | --- | --- | --- |
|  |  | Q1  (-2.74, -0.28) | Q2  (-0.28, -0.05) | Q3 (-0.05, 0.15) | Q4  (0.15, 0.38) | Q5  (0.38, 3.71) | *p*-value |
| N | 130,533 | 26,107 | 26,107 | 26,107 | 26,106 | 26,106 |  |
| Age, years | 33.42±5.7 | 33.61±5.68 | 33.51±5.65 | 33.52±5.68 | 33.44±5.71 | 33.02±5.77 | <0.001 |
| Alcohol intake$\geq1$0g/day (%) | 16,666 (14.04) | 3,381 (12.95) | 3,200 (12.26) | 3,222 (12.34) | 3,210 (12.3) | 3,653 (13.99) | <0.001 |
| Regular physical activity (%) | 11,791 (9.12) | 2,216 (8.49) | 2,213 (8.48) | 2,225 (8.52) | 2,357 (9.03) | 2,780 (10.65) | <0.001 |
| Smoking status (%) |  |  |  |  |  |  | <0.001 |
| Never | 115,134 (91.24) | 22,863 (87.57) | 23,109 (88.52) | 23,118 (88.55) | 23,166 (88.74) | 22,878 (87.64) |  |
| Former | 7,751 (6.14) | 1,602 (6.14) | 1,517 (5.81) | 1,484 (5.68) | 1,452 (5.56) | 1,696 (6.5) |  |
| Current | 3,302 (2.62) | 702 (2.69) | 613 (2.35) | 634 (2.43) | 673 (2.58) | 680 (2.6) |  |
| Education: University or more (%) | 87,013 (68.59) | 17,291 (66.23) | 17,814 (68.23) | 17,895 (68.54) | 17,607 (67.44) | 16,406 (62.84) | <0.001 |
| BMI, kg/m^2^ | 21.35±3.01 | 21.68±3.19 | 21.3±3.05 | 21.23±2.95 | 21.18±2.93 | 21.35±2.92 | <0.001 |
| Waist circumference, cm | 73.99±7.99 | 74.95±8.37 | 73.83±8.02 | 73.63±7.86 | 73.56±7.82 | 74.01±7.78 | <0.001 |
| SBP, mmHg | 101.35±9.96 | 102.27±10.29 | 101.4±9.92 | 101.16±9.93 | 100.93±9.88 | 100.98±9.69 | <0.001 |
| DBP, mmHg | 64.73±7.73 | 65.37±7.96 | 64.8±7.67 | 64.6±7.68 | 64.48±7.67 | 64.4±7.61 | <0.001 |
| Total cholesterol, mg/dL | 183.46±30.39 | 186.54±31.84 | 183.56±30.03 | 182.66±29.58 | 182.27±29.73 | 182.29±30.49 | <0.001 |
| HbA1c, % | 5.4±0.27 | 5.41±0.27 | 5.4±0.26 | 5.39±0.27 | 5.39±0.27 | 5.39±0.27 | <0.001 |
| Insulin, uIU/mL | 6.07±3.56 | 7.09±3.95 | 6.33±3.55 | 5.97±3.4 | 5.65±3.32 | 5.33±3.26 | <0.001 |
| eGFR, mL/min/1.73m^2^ | 112±11.75 | 112.24±11.85 | 112.01±11.66 | 111.85±11.67 | 111.84±11.68 | 112.07±11.87 | <0.001 |
| Uric acid, mg/dL | 4.3±0.9 | 4.28±0.91 | 4.26±0.88 | 4.27±0.89 | 4.31±0.88 | 4.41±0.93 | <0.001 |
| FBG, mg/dL | 89.7±7.5 | 91.77±7.25 | 90.55±7.03 | 89.79±7.14 | 88.79±7.4 | 87.59±7.93 | <0.001 |
| Triglycerides, mg/dL | 77.82±40.62 | 103.79±54.49 | 82.05±37.48 | 73.78±33.8 | 67.97±29.85 | 61.53±27.82 | <0.001 |
| TyG index |  |  |  |  |  |  |  |
| 1^st^ | 8.06±0.45 | 8.37±0.43 | 8.14±0.4 | 8.03±0.39 | 7.94±0.39 | 7.82±0.41 | <0.001 |
| 2^nd^ | 8.11±0.46 | 7.84±0.41 | 7.98±0.4 | 8.08±0.39 | 8.19±0.39 | 8.45±0.44 | <0.001 |
| HOMA-IR |  |  |  |  |  |  |  |
| 1^st^ | 1.37±0.87 | 1.63±0.98 | 1.44±0.87 | 1.35±0.83 | 1.26±0.8 | 1.18±0.78 | <0.001 |
| 2^nd^ | 1.47±0.95 | 1.21±0.81 | 1.34±0.87 | 1.44±0.9 | 1.54±0.93 | 1.81±1.1 | <0.001 |
| History of hypertension (%) | 1,563 (1.2) | 352 (1.35) | 292 (1.12) | 322 (1.23) | 282 (1.08) | 315 (1.21) | 0.045 |
| Usage of lipid-lowering medication (%) | 432 (0.33) | 93 (0.36) | 93 (0.36) | 73 (0.28) | 70 (0.27) | 103 (0.39) | 0.051 |
| Family history of diabetes (%) | 17,828 (13.66) | 3,638 (13.93) | 3,495 (13.39) | 3,459 (13.25) | 3,575 (13.69) | 3,661 (14.02) | 0.040 |
| Menopause (%) | 1,928 (1.56) | 400 (1.53) | 339 (1.3) | 403 (1.54) | 374 (1.43) | 412 (1.58) | 0.073 |

**Abbreviations**: BMI, body mass index; ; DBP, diastolic blood pressure; eGFR, estimated Glomerular Filtration Rate, FBG, fasting blood glucose; HbA1c, glycated hemoglobin; HOMA-IR, homeostasis model assessment of insulin resistance; SBP, systolic blood pressure; TyG index, triglyceride-glucose index; Q, quintile.

Data are expressed as mean$\pm$standard deviation or a number (percentage). One-way ANOVA was used for continuous variables and chi-square test for categorical variables.

Alcohol intake was defined as ≥20 g/day for men and ≥10 g/day for women. Regular physical activity defined as vigorous exercise frequency $\geq$3times/week.

**Supplementary Table S5. Baseline Characteristics of Women by Quintiles of HOMA-IR Change**

|  | Total | HOMA-IR change quintiles | | | | | |
| --- | --- | --- | --- | --- | --- | --- | --- |
|  |  | Q1  (-7.20, -0.49) | Q2  (-0.49, -0.09) | Q3 (-0.09, 0.23) | Q4  (0.23, 0.66) | Q5  (0.66, 8.46) | *p*-value |
| N | 130,533 | 26,107 | 26,107 | 26,107 | 26,106 | 26,106 |  |
| Age, years | 33.42±5.7 | 33.29±5.82 | 33.78±5.69 | 33.76±5.67 | 33.49±5.6 | 32.77±5.66 | <0.001 |
| Alcohol intake$\geq1$0g/day (%) | 16,666 (14.04) | 3,366 (12.89) | 3,329 (12.75) | 3,209 (12.29) | 3,327 (12.74) | 3,435 (13.16) | 0.073 |
| Regular physical activity (%) | 11,791 (9.12) | 2,075 (7.95) | 2,242 (8.59) | 2,427 (9.3) | 2,519 (9.65) | 2,528 (9.68) | <0.001 |
| Smoking status (%) |  |  |  |  |  |  | 0.025 |
| Never | 115,134 (91.24) | 23,142 (88.64) | 23,025 (88.19) | 23,033 (88.23) | 22,998 (88.09) | 22,936 (87.86) |  |
| Former | 7,751 (6.14) | 1,603 (6.14) | 1,490 (5.71) | 1,506 (5.77) | 1,521 (5.83) | 1,631 (6.25) |  |
| Current | 3,302 (2.62) | 649 (2.49) | 642 (2.46) | 636 (2.44) | 650 (2.49) | 725 (2.78) |  |
| Education: University or more (%) | 87,013 (68.59) | 17,228 (65.99) | 17,802 (68.19) | 17,832 (68.3) | 17,890 (68.53) | 16,261 (62.29) | <0.001 |
| BMI, kg/m^2^ | 21.35±3.01 | 22±3.41 | 21.01±2.68 | 20.85±2.63 | 20.98±2.7 | 21.91±3.34 | <0.001 |
| Waist circumference, cm | 73.99±7.99 | 75.52±8.75 | 73.13±7.32 | 72.74±7.21 | 73.13±7.4 | 75.45±8.64 | <0.001 |
| SBP, mmHg | 101.35±9.96 | 103.21±10.31 | 100.99±9.64 | 100.34±9.61 | 100.38±9.68 | 101.82±10.24 | <0.001 |
| DBP, mmHg | 64.73±7.73 | 65.85±7.85 | 64.66±7.58 | 64.16±7.56 | 64.17±7.65 | 64.81±7.87 | <0.001 |
| Total cholesterol, mg/dL | 183.46±30.39 | 185±30.81 | 182.97±29.83 | 182.83±30.08 | 182.79±30.23 | 183.72±30.92 | <0.001 |
| HbA1c, % | 5.4±0.27 | 5.4±0.27 | 5.39±0.26 | 5.39±0.27 | 5.39±0.27 | 5.4±0.27 | <0.001 |
| Insulin, uIU/mL | 6.07±3.56 | 9.37±4.19 | 5.97±2.56 | 4.97±2.52 | 4.7±2.62 | 5.36±3.37 | <0.001 |
| eGFR, mL/min/1.73m^2^ | 112±11.75 | 112.33±11.82 | 111.59±11.75 | 111.61±11.67 | 111.81±11.69 | 112.68±11.77 | <0.001 |
| Uric acid, mg/dL | 4.3±0.9 | 4.31±0.92 | 4.2±0.86 | 4.24±0.87 | 4.31±0.88 | 4.47±0.95 | <0.001 |
| FBG, mg/dL | 89.7±7.5 | 93.6±7.1 | 90.42±6.59 | 88.74±7 | 87.83±7.27 | 87.91±7.88 | <0.001 |
| Triglycerides, mg/dL | 77.82±40.62 | 90.09±48.86 | 77.12±38.33 | 72.94±35.1 | 71.65±34.76 | 77.31±41.74 | <0.001 |
| TyG index |  |  |  |  |  |  |  |
| 1^st^ | 8.06±0.45 | 8.24±0.45 | 8.07±0.41 | 7.99±0.42 | 7.97±0.42 | 8.02±0.47 | <0.001 |
| 2^nd^ | 8.11±0.46 | 8.03±0.46 | 8.01±0.42 | 8.05±0.42 | 8.13±0.42 | 8.33±0.47 | <0.001 |
| HOMA-IR |  |  |  |  |  |  |  |
| 1^st^ | 1.37±0.87 | 2.18±1.05 | 1.34±0.62 | 1.1±0.6 | 1.04±0.62 | 1.19±0.81 | <0.001 |
| 2^nd^ | 1.47±0.95 | 1.15±0.75 | 1.07±0.61 | 1.17±0.6 | 1.47±0.63 | 2.48±1.2 | <0.001 |
| History of hypertension (%) | 1,563 (1.2) | 367 (1.41) | 275 (1.05) | 265 (1.02) | 295 (1.13) | 361 (1.38) | <0.001 |
| Usage of lipid-lowering medication (%) | 432 (0.33) | 105 (0.4) | 74 (0.28) | 64 (0.25) | 69 (0.26) | 120 (0.46) | <0.001 |
| Family history of diabetes (%) | 17,828 (13.66) | 3,537 (13.55) | 3,561 (13.64) | 3,510 (13.44) | 3,579 (13.71) | 3,641 (13.95) | 0.527 |
| Menopause (%) | 1,928 (1.56) | 340 (1.3) | 403 (1.54) | 396 (1.52) | 388 (1.49) | 401 (1.54) | 0.102 |

**Abbreviations**: BMI, body mass index; ; DBP, diastolic blood pressure; eGFR, estimated Glomerular Filtration Rate, FBG, fasting blood glucose; HbA1c, glycated hemoglobin; HOMA-IR, homeostasis model assessment of insulin resistance; SBP, systolic blood pressure; TyG index, triglyceride-glucose index; Q, quintile.

Data are expressed as mean$\pm$standard deviation or a number (percentage). One-way ANOVA was used for continuous variables and chi-square test for categorical variables.

Alcohol intake was defined as ≥20 g/day for men and ≥10 g/day for women. Regular physical activity defined as vigorous exercise frequency $\geq$3times/week.

**Supplementary Table S6. Risk of incident T2DM stratified by family history of diabetes in men**

|  | Quintile | Events (n) | Duration (PY) | Incidence rate  (per 10^3^ PY) | Age-adjusted HR (95% CI) | Multivariable-adjusted HR (95% CI) | |
| --- | --- | --- | --- | --- | --- | --- | --- |
|  |  |  |  |  |  | Model 1 | Model 2 |
| TyG index | **(1) Family History of Diabetes (No)** | | | | | | |
|  | Q1 | 613 | 93,216.6 | 6.6 | 1.03 (0.92, 1.15) | 0.69 (0.61, 0.78) | 0.70 (0.62, 0.79) |
|  | Q2 | 551 | 95,966.2 | 5.7 | 0.91 (0.81, 1.02) | 0.81 (0.72, 0.92) | 0.81 (0.72, 0.92) |
|  | Q3 | 615 | 98,595.2 | 6.2 | reference | reference | reference |
|  | Q4 | 624 | 99,171.9 | 6.3 | 1.03 (0.92, 1.15) | 1.22 (1.08, 1.37) | 1.22 (1.09, 1.37) |
|  | Q5 | 661 | 96,338.8 | 6.9 | 1.19 (1.06, 1.32) | 1.72 (1.53, 1.93) | 1.72 (1.53, 1.93) |
|  | **Total** | 125,525 |  | *p* for trend | 0.007 | <0.001 | <0.001 |
|  | **(2) Family History of Diabetes (Yes)** | | | | | | |
|  | Q1 | 153 | 11,566.5 | 13.2 | 1.05 (0.84, 1.31) | 0.73 (0.58, 0.92) | 0.75 (0.60, 0.95) |
|  | Q2 | 153 | 11,767.2 | 13.0 | 1.02 (0.82, 1.28) | 0.89 (0.71, 1.12) | 0.88 (0.70, 1.11) |
|  | Q3 | 152 | 12,005.7 | 12.7 | reference | reference | reference |
|  | Q4 | 149 | 12,360.4 | 12.1 | 0.98 (0.78, 1.22) | 1.18 (0.93, 1.49) | 1.19 (0.94, 1.50) |
|  | Q5 | 170 | 12,037.6 | 14.1 | 1.20 (0.96, 1.49) | 1.73 (1.37, 2.17) | 1.74 (1.38, 2.20) |
|  | **Total** | 15,534 |  | *p* for trend | 0.233 | <0.001 | <0.001 |
| HOMA-IR | **(3) Family History of Diabetes (No)** | | | | | | |
|  | Q1 | 659 | 88,048.8 | 7.5 | 1.67 (1.48, 1.88) | 0.70 (0.61, 0.80) | 0.70 (0.61, 0.80) |
|  | Q2 | 457 | 96,812.9 | 4.7 | 1.02 (0.89, 1.16) | 0.85 (0.74, 0.97) | 0.85 (0.74, 0.97) |
|  | Q3 | 464 | 101,144.4 | 4.6 | reference | reference | reference |
|  | Q4 | 537 | 100,835.9 | 5.3 | 1.19 (1.05, 1.34) | 1.14 (1.01, 1.30) | 1.14 (1.00, 1.29) |
|  | Q5 | 947 | 96,446.6 | 9.8 | 2.32 (2.07, 2.59) | 1.67 (1.49, 1.87) | 1.63 (1.45, 1.83) |
|  | **Total** | 125,525 |  | *p* for trend | <0.001 | <0.001 | <0.001 |
|  | **(4) Family History of Diabetes (Yes)** | | | | | | |
|  | Q1 | 164 | 10,932.3 | 15.0 | 1.60 (1.27, 2.03) | 0.75 (0.58, 0.98) | 0.76 (0.59, 0.99) |
|  | Q2 | 116 | 12,228.6 | 9.5 | 0.99 (0.76, 1.27) | 0.86 (0.66, 1.12) | 0.86 (0.66, 1.12) |
|  | Q3 | 120 | 12,387.5 | 9.7 | reference | reference | reference |
|  | Q4 | 155 | 12,637.5 | 12.3 | 1.29 (1.02, 1.64) | 1.27 (0.99, 1.62) | 1.26 (0.98, 1.61) |
|  | Q5 | 222 | 11,551.4 | 19.2 | 2.15 (1.72, 2.68) | 1.58 (1.25, 1.99) | 1.55 (1.23, 1.96) |
|  | **Total** | 15,534 |  | *p* for trend | <0.001 | <0.001 | <0.001 |

**Abbreviations**: BMI, body mass index; ; DBP, diastolic blood pressure; eGFR, estimated Glomerular Filtration Rate, FBG, fasting blood glucose; HbA1c, glycated hemoglobin; HOMA-IR, homeostasis model assessment of insulin resistance; SBP, systolic blood pressure; TyG index, triglyceride-glucose index; Q, quintile.

Median (IQR) of follow-up duration: (1)&(3) 3.18 (6.41) years, (2)&(4) 2.98 (6.5) years.

Model 1 was adjusted for age, examination center, body mass index, education, smoking status, alcohol intake, regular physical activity, history of hypertension, use of lipid-lowering medication, eGFR, baseline TyG index, and baseline HOMA-IR.
Model 2 was adjusted for variables in Model 1 and total cholesterol, high-density lipoprotein cholesterol, low-density lipoprotein cholesterol, and uric acid.**Supplementary Table S7. Risk of incident Type 2 Diabetes stratified by family history of diabetes in women**

|  | Quintile | Events (n) | Duration (PY) | Incidence rate  (per 10^3^ PY) | Age-adjusted HR (95% CI) | Multivariable-adjusted HR (95% CI) | |
| --- | --- | --- | --- | --- | --- | --- | --- |
|  |  |  |  |  |  | Model 1 | Model 2 |
| TyG index | **(1) Family History (No)** | | | | | | |
|  | Q1 | 148 | 85,054.2 | 1.7 | 1.17 (0.92, 1.48) | 0.56 (0.43, 0.72) | 0.58 (0.45, 0.76) |
|  | Q2 | 134 | 86,366.1 | 1.6 | 1.04 (0.81, 1.32) | 0.82 (0.63, 1.06) | 0.82 (0.63, 1.06) |
|  | Q3 | 130 | 87,080.3 | 1.5 | reference | reference | reference |
|  | Q4 | 145 | 87,613.8 | 1.7 | 1.12 (0.88, 1.42) | 1.31 (1.02, 1.69) | 1.31 (1.02, 1.69) |
|  | Q5 | 208 | 86,920.0 | 2.4 | 1.68 (1.35, 2.10) | 2.19 (1.72, 2.78) | 2.12 (1.67, 2.70) |
|  | **Total** | 112,705 |  | *p* for trend | <0.001 | <0.001 | <0.001 |
|  | **(2) Family History (Yes)** | | | | | | |
|  | Q1 | 63 | 13,470.9 | 4.7 | 1.12 (0.78, 1.61) | 0.71 (0.47, 1.08) | 0.71 (0.47, 1.09) |
|  | Q2 | 51 | 13,783.0 | 3.7 | 0.87 (0.59, 1.27) | 0.80 (0.52, 1.23) | 0.78 (0.51, 1.21) |
|  | Q3 | 56 | 13,345.2 | 4.2 | reference | reference | reference |
|  | Q4 | 49 | 13,437.0 | 3.6 | 0.89 (0.61, 1.31) | 1.24 (0.81, 1.92) | 1.21 (0.78, 1.86) |
|  | Q5 | 85 | 13,666.2 | 6.2 | 1.58 (1.12, 2.21) | 2.35 (1.60, 3.45) | 2.23 (1.52, 3.27) |
|  | **Total** | 17,828 |  | *p* for trend | 0.042 | <0.001 | <0.001 |
| HOMA-IR | **(3) Family History (No)** | | | | | | |
|  | Q1 | 176 | 78,761.2 | 2.2 | 2.21 (1.72, 2.83) | 0.75 (0.57, 1.00) | 0.77 (0.58, 1.03) |
|  | Q2 | 113 | 87,366.3 | 1.3 | 1.21 (0.92, 1.59) | 1.07 (0.80, 1.43) | 1.07 (0.80, 1.44) |
|  | Q3 | 97 | 90,661.8 | 1.1 | reference | reference | reference |
|  | Q4 | 108 | 90,254.4 | 1.2 | 1.14 (0.87, 1.50) | 1.19 (0.89, 1.60) | 1.17 (0.87, 1.57) |
|  | Q5 | 271 | 85,990.6 | 3.2 | 3.20 (2.53, 4.03) | 2.04 (1.58, 2.63) | 1.96 (1.52, 2.53) |
|  | **Total** | 112,705 |  | *p* for trend | <0.001 | <0.001 | <0.001 |
|  | **(4) Family History (Yes)** | | | | | | |
|  | Q1 | 73 | 12,422.3 | 5.9 | 2.53 (1.68, 3.80) | 1.38 (0.83, 2.27) | 1.36 (0.83, 2.25) |
|  | Q2 | 45 | 13,668.6 | 3.3 | 1.34 (0.86, 2.09) | 1.32 (0.79, 2.22) | 1.31 (0.78, 2.21) |
|  | Q3 | 34 | 13,980.5 | 2.4 | reference | reference | reference |
|  | Q4 | 45 | 14,098.7 | 3.2 | 1.34 (0.86, 2.09) | 1.44 (0.86, 2.42) | 1.38 (0.82, 2.33) |
|  | Q5 | 107 | 13,532.2 | 7.9 | 3.61 (2.45, 5.32) | 2.90 (1.84, 4.58) | 2.69 (1.70, 4.26) |
|  | **Total** | 17,828 |  | *p* for trend | <0.001 | <0.001 | <0.001 |

**Abbreviations:** CI, confidence interval; HOMA-IR, homeostasis model assessment of insulin resistance; HR, hazard ratio; PY, person-year; Q, quintile; TyG index, triglyceride-glucose index.

Median (IQR) of follow-up duration: (1)&(3) 3.3 (6.38) years, (2)&(4) 3(6.47) years
Model 1 was adjusted for age, examination center, body mass index, education, smoking status, alcohol intake, regular physical activity, history of hypertension, use of lipid-lowering medication, eGFR, baseline TyG index, baseline HOMA-IR, and menopause (for women).

Model 2 was adjusted for the variables in Model 1, total cholesterol, high-density lipoprotein cholesterol, low-density lipoprotein cholesterol, and uric acid.

**Supplementary Table S8. Baseline comparison: excluded (HOMA-IR≥10) vs. included participants**

| **Variables** | **Men** | | | **Women** | | |
| --- | --- | --- | --- | --- | --- | --- |
|  | Excluded (≥10) | Included (<10) | *p*-value | Excluded (≥10) | Included (<10) | *p*-value |
| N | 96 | 142,172 |  | 59 | 131,044 |  |
| Age, years | 32.53 ± 5.98 | 34.10 ± 5.57 | 0.012 | 30.24 ± 5.19 | 33.41 ± 5.70 | <0.001 |
| Fasting insulin, uIU/mL | 50.15 ± 36.95 | 7.00 ± 4.22 | <0.001 | 73.82 ± 120.61 | 6.11 ± 3.64 | <0.001 |
| FBG, mg/dL | 107.32 ± 9.36 | 93.89 ± 7.98 | <0.001 | 103.20 ± 11.15 | 89.72 ± 7.51 | <0.001 |
| BMI, kg/m^2^ | 32.39±4.58 | 24.68±3.11 | <0.001 | 31.32 ± 5.62 | 21.38 ± 3.06 | <0.001 |
| SBP, mmHg | 126.30 ± 12.81 | 114.00 ± 10.91 | <0.001 | 118.05 ± 16.61 | 101.39 ± 9.99 | <0.001 |
| Triglycerides, mg/dL | 217.15 ± 165.83 | 125.81 ± 84.20 | <0.001 | 182.22 ± 142.21 | 78.02 ± 40.92 | <0.001 |
| History of hypertension (%) | 30 (31.2) | 10,496 (7.4) | <0.001 | 4 (6.8) | 1,598 (1.2) | 0.001 |
| Family history of diabetes (%) | 11 (11.5) | 15,683 (11.0) | 1.000 | 9 (15.3) | 17,927 (13.7) | 0.871 |

**Abbreviations:** BMI, body mass index; FBG, fasting blood glucose; HOMA-IR, homeostasis model assessment of insulin resistance; SBP, systolic blood pressure.

**Supplementary Table S9. Sensitivity analyses according to different approaches to handling extreme baseline HOMA-IR values**

|  | Quintile | Main analysis | All HOMA-IR values included | HOMA-IR Winsorized  (1^st^-99^th^ percentile) |
| --- | --- | --- | --- | --- |
| Male | TyG index |  |  |  |
|  | Q1 | 0.71 (0.64, 0.79) | 0.67 (0.61, 0.74) | 0.68 (0.61, 0.74) |
|  | Q2 | 0.81 (0.73, 0.90) | 0.80 (0.72, 0.88) | 0.79 (0.72, 0.88) |
|  | Q3 (Reference) | reference | reference | reference |
|  | Q4 | 1.20 (1.08, 1.33) | 1.15 (1.05, 1.27) | 1.16 (1.05, 1.27) |
|  | Q5 | 1.72 (1.55, 1.91) | 1.63 (1.48, 1.80) | 1.67 (1.51, 1.83) |
|  | HOMA-IR |  |  |  |
|  | Q1 | 0.72 (0.64, 0.81) | 0.77 (0.69, 0.86) | 0.68 (0.62, 0.76) |
|  | Q2 | 0.85 (0.76, 0.96) | 0.86 (0.77, 0.97) | 0.86 (0.77, 0.97) |
|  | Q3 (Reference) | reference | reference | reference |
|  | Q4 | 1.16 (1.03, 1.30) | 1.17 (1.05, 1.31) | 1.21 (1.09, 1.35) |
|  | Q5 | 1.63 (1.47, 1.81) | 1.70 (1.54, 1.88) | 1.75 (1.59, 1.93) |
| Female | TyG index |  |  |  |
|  | Q1 | 0.60 (0.48, 0.76) | 0.54 (0.44, 0.66) | 0.57 (0.47, 0.70) |
|  | Q2 | 0.81 (0.65, 1.01) | 0.77 (0.63, 0.94) | 0.78 (0.64, 0.96) |
|  | Q3 (Reference) | reference | reference | reference |
|  | Q4 | 1.28 (1.03, 1.60) | 1.19 (0.98, 1.45) | 1.20 (0.99, 1.46) |
|  | Q5 | 2.18 (1.78, 2.67) | 2.12 (1.77, 2.54) | 2.16 (1.80, 2.58) |
|  | HOMA-IR |  |  |  |
|  | Q1 | 0.92 (0.71, 1.17) | 1.21 (0.97, 1.52) | 0.88 (0.71, 1.09) |
|  | Q2 | 1.14 (0.89, 1.47) | 1.13 (0.89, 1.44) | 1.16 (0.92, 1.47) |
|  | Q3 (Reference) | reference | reference | reference |
|  | Q4 | 1.23 (0.96, 1.60) | 1.17 (0.92, 1.49) | 1.37 (1.08, 1.72) |
|  | Q5 | 2.14 (1.71, 2.67) | 2.22 (1.81, 2.73) | 2.45 (2.01, 2.99) |

**Abbreviations:** eGFR, estimated glomerular filtration rate; HOMA-IR, homeostasis model assessment of insulin resistance; TyG index, triglyceride-glucose index; Q, quintile.

All models were based on Cox proportional hazards regression and adjusted for age, examination center, body mass index, education, smoking status, alcohol intake, regular physical activity, history of hypertension, use of lipid-lowering medication, eGFR, baseline TyG index, baseline HOMA-IR, total cholesterol, high-density lipoprotein cholesterol, low-density lipoprotein cholesterol, uric acid, and menopause (for women).

The main analysis excluded participants with extreme baseline HOMA-IR values (≥10). In sensitivity analyses, (1) all baseline HOMA-IR values were included, and (2) HOMA-IR values were winsorized at the 1st and 99th percentiles.

**Supplementary Table S10. Sensitivity analyses with additional adjustment for second-examination covariates**

|  | Quintile | Main analysis | Additional adjustment for second exam covariates |
| --- | --- | --- | --- |
| Male | TyG index |  |  |
|  | Q1 | 0.71 (0.64, 0.79) | 0.81 (0.73, 0.90) |
|  | Q2 | 0.81 (0.73, 0.90) | 0.85 (0.76, 0.94) |
|  | Q3 (Reference) | reference | reference |
|  | Q4 | 1.20 (1.08, 1.33) | 1.15 (1.04, 1.28) |
|  | Q5 | 1.72 (1.55, 1.91) | 1.56 (1.40, 1.73) |
|  | HOMA-IR |  |  |
|  | Q1 | 0.72 (0.64, 0.81) | 0.79 (0.70, 0.89) |
|  | Q2 | 0.85 (0.76, 0.96) | 0.87 (0.77, 0.98) |
|  | Q3 (Reference) | reference | reference |
|  | Q4 | 1.16 (1.03, 1.30) | 1.11 (0.99, 1.24) |
|  | Q5 | 1.63 (1.47, 1.81) | 1.47 (1.32, 1.63) |
| Female | TyG index |  |  |
|  | Q1 | 0.60 (0.48, 0.76) | 0.73 (0.58, 0.93) |
|  | Q2 | 0.81 (0.65, 1.01) | 0.82 (0.64, 1.03) |
|  | Q3 (Reference) | reference | reference |
|  | Q4 | 1.28 (1.03, 1.60) | 1.19 (0.94, 1.50) |
|  | Q5 | 2.18 (1.78, 2.67) | 1.92 (1.54, 2.38) |
|  | HOMA-IR |  |  |
|  | Q1 | 0.92 (0.71, 1.17) | 1.05 (0.81, 1.36) |
|  | Q2 | 1.14 (0.89, 1.47) | 1.11 (0.85, 1.45) |
|  | Q3 (Reference) | reference | reference |
|  | Q4 | 1.23 (0.96, 1.60) | 1.13 (0.87, 1.48) |
|  | Q5 | 2.14 (1.71, 2.67) | 1.78 (1.41, 2.26) |

**Abbreviations:** eGFR, estimated glomerular filtration rate; HOMA-IR, homeostasis model assessment of insulin resistance; Q, quintile; TyG index, triglyceride-glucose index.

Main analysis was based on Cox proportional hazards regression and adjusted for age, examination center, body mass index, education, smoking status, alcohol intake, regular physical activity, history of hypertension, use of lipid-lowering medication, eGFR, baseline TyG index, baseline HOMA-IR, total cholesterol, high-density lipoprotein cholesterol, low-density lipoprotein cholesterol, uric acid, and menopause (for women) at the baseline examination.

In the additional adjustment model, selected cardiometabolic covariates (body mass index, systolic and diastolic blood pressure, smoking status, alcohol intake, and lipid-lowering medication use) were replaced with corresponding values measured at the second examination, while all other covariates were retained as in the main model.

**Supplementary Table S11. Sensitivity analyses modeling changes in the TyG index and HOMA-IR as continuous variables**

| Sex | Change variable | HR (95% CI) | *P*-value |
| --- | --- | --- | --- |
| Male | TyG index | 1.40 (1.35, 1.45) | <0.001 |
|  | HOMA-IR | 1.31 (1.27, 1.34) | <0.001 |
| Female | TyG index | 1.56 (1.46, 1.66) | <0.001 |
|  | HOMA-IR | 1.31 (1.25, 1.37) | <0.001 |

**Abbreviations:** CI, confidence interval; eGFR, estimated glomerular filtration rate; HOMA-IR, homeostasis model assessment of insulin resistance; HR, hazard ratio; TyG index, triglyceride-glucose index.

Hazard ratios (HRs) were estimated per 1–standard deviation increase in changes in the TyG index or HOMA-IR.

Model was adjusted for age, examination center, body mass index, education, smoking status, alcohol intake, regular physical activity, history of hypertension, use of lipid-lowering medication, eGFR, baseline TyG index, baseline HOMA-IR, menopause (for women), total cholesterol, high-density lipoprotein cholesterol, low-density lipoprotein cholesterol, and uric acid.

**Supplementary Table S12. Sensitivity analyses additionally adjusting for the interval between the first and second examinations**

| Sex | Change variable | HR (95% CI) | *P*-value |
| --- | --- | --- | --- |
| Male | TyG index | 1.14 (1.09, 1.18) | <0.001 |
|  | HOMA-IR | 1.14 (1.10, 1.18) | <0.001 |
| Female | TyG index | 1.25 (1.17, 1.33) | <0.001 |
|  | HOMA-IR | 1.27 (1.19, 1.35) | <0.001 |

**Abbreviations:** CI, confidence interval; eGFR, estimated glomerular filtration rate; HOMA-IR, homeostasis model assessment of insulin resistance; HR, hazard ratio; TyG index, triglyceride-glucose index.

Hazard ratios (HRs) were estimated per 1–standard deviation increase in changes in the TyG index or HOMA-IR using Cox proportional hazards models, with additional adjustment for the interval between the first and second examinations.

Model was adjusted for age, examination center, body mass index, education, smoking status, alcohol intake, regular physical activity, history of hypertension, use of lipid-lowering medication, eGFR, baseline TyG index, baseline HOMA-IR, menopause (for women), total cholesterol, high-density lipoprotein cholesterol, low-density lipoprotein cholesterol, and uric acid.

**Supplementary Table S13. Sensitivity analyses addressing missing data and potential selection bias**

|  | Quintile | Primary complete-case model | Multiple imputation model | Inverse probability weighted model |
| --- | --- | --- | --- | --- |
| Male | TyG index |  |  |  |
|  | Q1 | 0.71 (0.64, 0.79) | 0.73 (0.66, 0.81) | 0.72 (0.65, 0.80) |
|  | Q2 | 0.81 (0.73, 0.90) | 0.85 (0.77, 0.94) | 0.82 (0.74, 0.92) |
|  | Q3 (Reference) | reference | reference | reference |
|  | Q4 | 1.20 (1.08, 1.33) | 1.25 (1.14, 1.38) | 1.21 (1.09, 1.35) |
|  | Q5 | 1.72 (1.55, 1.91) | 1.65 (1.50, 1.82) | 1.73 (1.55, 1.92) |
|  | HOMA-IR |  |  |  |
|  | Q1 | 0.72 (0.64, 0.81) | 0.75 (0.67, 0.83) | 0.72 (0.64, 0.82) |
|  | Q2 | 0.85 (0.76, 0.96) | 0.85 (0.76, 0.95) | 0.85 (0.75, 0.96) |
|  | Q3 (Reference) | reference | reference | reference |
|  | Q4 | 1.16 (1.03, 1.30) | 1.17 (1.06, 1.30) | 1.15 (1.03, 1.30) |
|  | Q5 | 1.63 (1.47, 1.81) | 1.58 (1.44, 1.74) | 1.61 (1.45, 1.79) |
| Female | TyG index |  |  |  |
|  | Q1 | 0.60 (0.48, 0.76) | 0.58 (0.47, 0.72) | 0.63 (0.49, 0.79) |
|  | Q2 | 0.81 (0.65, 1.01) | 0.79 (0.64, 0.98) | 0.82 (0.65, 1.03) |
|  | Q3 (Reference) | reference | reference | reference |
|  | Q4 | 1.28 (1.03, 1.60) | 1.23 (1.00, 1.50) | 1.29 (1.03, 1.62) |
|  | Q5 | 2.18 (1.78, 2.67) | 2.07 (1.72, 2.50) | 2.18 (1.76, 2.70) |
|  | HOMA-IR |  |  |  |
|  | Q1 | 0.92 (0.71, 1.17) | 0.87 (0.69, 1.09) | 0.97 (0.75, 1.26) |
|  | Q2 | 1.14 (0.89, 1.47) | 1.07 (0.85, 1.35) | 1.13 (0.87, 1.46) |
|  | Q3 (Reference) | reference | reference | reference |
|  | Q4 | 1.23 (0.96, 1.60) | 1.17 (0.93, 1.48) | 1.25 (0.96, 1.62) |
|  | Q5 | 2.14 (1.71, 2.67) | 2.09 (1.71, 2.57) | 2.12 (1.68, 2.68) |

**Abbreviations:** HOMA-IR, homeostasis model assessment of insulin resistance; TyG index, triglyceride-glucose index.

The primary model corresponds to Model 2 in the main analysis (complete-case analysis). Multiple imputation was performed using chained equations including exposure, outcome, and all covariates.

Inverse probability weighting accounted for the probability of having at least two health examinations.

**Supplementary Table S14. Sensitivity analyses using residual change scores and joint modeling of baseline and follow-up values**

|  | Quintile | Main analysis | Residual change | Joint modeling |
| --- | --- | --- | --- | --- |
| Male | TyG index |  |  |  |
|  | Q1 | 0.71 (0.64, 0.79) | 0.74 (0.66, 0.84) | 0.67 (0.57, 0.79) |
|  | Q2 | 0.81 (0.73, 0.90) | 0.86 (0.77, 0.96) | 0.87 (0.76, 1.00) |
|  | Q3 (Reference) | reference | reference | reference |
|  | Q4 | 1.20 (1.08, 1.33) | 1.23 (1.11, 1.36) | 1.40 (1.25, 1.56) |
|  | Q5 | 1.72 (1.55, 1.91) | 1.64 (1.49, 1.81) | 1.94 (1.74, 2.17) |
|  | HOMA-IR |  |  |  |
|  | Q1 | 0.72 (0.64, 0.81) | 0.80 (0.71, 0.91) | 0.71 (0.61, 0.82) |
|  | Q2 | 0.85 (0.76, 0.96) | 0.86 (0.76, 0.97) | 0.78 (0.68, 0.89) |
|  | Q3 (Reference) | reference | reference | reference |
|  | Q4 | 1.16 (1.03, 1.30) | 1.29 (1.15, 1.44) | 1.31 (1.18, 1.47) |
|  | Q5 | 1.63 (1.47, 1.81) | 1.76 (1.59, 1.95) | 1.98 (1.78, 2.20) |
| Female | TyG index |  |  |  |
|  | Q1 | 0.60 (0.48, 0.76) | 0.65 (0.51, 0.83) | 0.52 (0.36, 0.73) |
|  | Q2 | 0.81 (0.65, 1.01) | 0.76 (0.59, 0.97) | 0.66 (0.48, 0.90) |
|  | Q3 (Reference) | reference | reference | reference |
|  | Q4 | 1.28 (1.03, 1.60) | 1.23 (0.99, 1.52) | 1.17 (0.91, 1.49) |
|  | Q5 | 2.18 (1.78, 2.67) | 1.98 (1.64, 2.40) | 2.36 (1.88, 2.96) |
|  | HOMA-IR |  |  |  |
|  | Q1 | 0.92 (0.71, 1.17) | 0.90 (0.70, 1.17) | 0.70 (0.49, 0.99) |
|  | Q2 | 1.14 (0.89, 1.47) | 1.09 (0.84, 1.43) | 1.10 (0.81, 1.49) |
|  | Q3 (Reference) | reference | reference | reference |
|  | Q4 | 1.23 (0.96, 1.60) | 1.24 (0.96, 1.59) | 1.82 (1.40, 2.37) |
|  | Q5 | 2.14 (1.71, 2.67) | 2.30 (1.84, 2.87) | 3.09 (2.42, 3.95) |

**Abbreviations:** eGFR, estimated glomerular filtration rate; HOMA-IR, homeostasis model assessment of insulin resistance; TyG index, triglyceride-glucose index; Q, quintile.

All models were based on Cox proportional hazards regression and adjusted for age, examination center, body mass index, education, smoking status, alcohol intake, regular physical activity, history of hypertension, use of lipid-lowering medication, eGFR, baseline TyG index, baseline HOMA-IR, total cholesterol, high-density lipoprotein cholesterol, low-density lipoprotein cholesterol, uric acid, and menopause (for women).

Residual change scores were derived from the residuals of a linear regression of the follow-up value on the corresponding baseline value. Joint modeling included both baseline and follow-up values simultaneously as exposures in the Cox proportional hazards model.


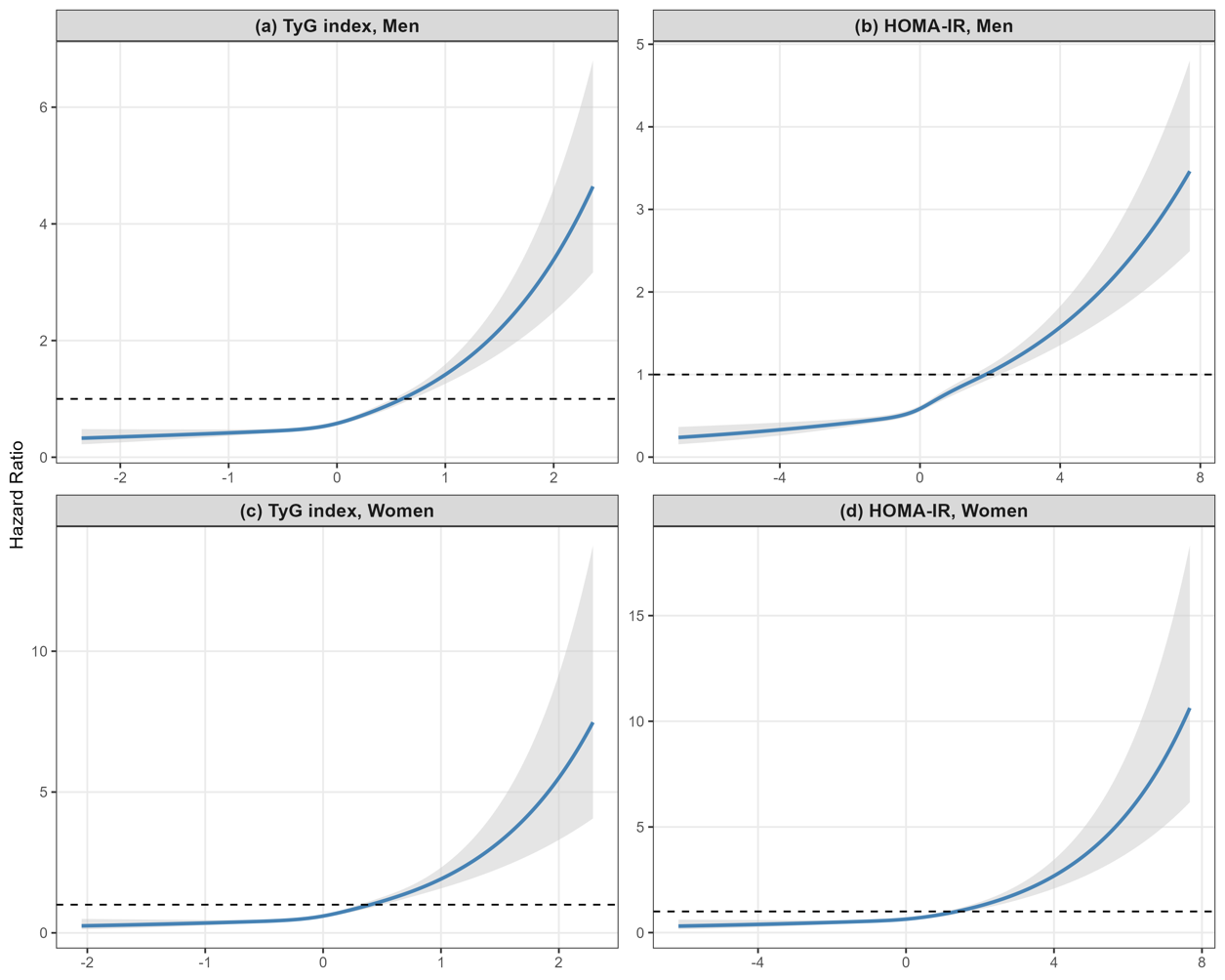


**Supplementary Figure S1. Restricted cubic spline analyses of changes in TyG index and HOMA-IR in relation to incident type 2 diabetes.** HRs (solid lines) and 95% CIs (shaded areas) were estimated using Cox proportional hazards models adjusted for covariates in Model 2. The median value of each index change was used as the reference point. (a) Change in TyG index, men. (b) Change in HOMA-IR, men. (c) Change in TyG index, women. (d) Change in HOMA-IR, women. Tests for non-linearity: men, TyG *P* < 0.001, HOMA-IR *P* = 0.014; women, TyG *P* = 0.001, HOMA-IR *P* = 0.001.
